# ALYREF-mediated m^5^C modification of CCNA1 drives escape from cell-cycle arrest and contributes to pazopanib resistance in Renal Cell Carcinoma

**DOI:** 10.7150/ijbs.131368

**Published:** 2026-06-17

**Authors:** Zeyi Lu, Yang Li, Ziwei Zhu, Fan Li, Yiming Ding, Lifeng Ding, Ruyue Wang, Yudong Lin, Wenqin Luo, Xudong Mao, Haohua Lu, Yejinpeng Wang, Mengxuan Li, Yuanlei Chen, Zhehao Xu, Yi Lu, Qiming Zheng, Haiyun Xie, Zhenwei Zhou, Liqun Xia, Gonghui Li, Mingchao Wang

**Affiliations:** Department of Urology, Sir Run Run Shaw Hospital, Zhejiang University School of Medicine, Hangzhou, 310016, China.

**Keywords:** pazopanib resistance, renal cell carcinoma, ALYREF, senescence-like phenotype, cell cycle

## Abstract

Pazopanib, a first-line tyrosine kinase inhibitor for advanced renal cell carcinoma (RCC), faces significant clinical limitations due to acquired resistance. In this study, we reveal a novel mechanism underlying pazopanib resistance in RCC, driven by a senescence-like phenotype without cell-cycle arrest. Transcriptomic profiling identified ALYREF as a key upregulated m^5^C reader in pazopanib resistant cells. Mechanistically, ALYREF stabilizes CCNA1 mRNA via m^5^C modification, promoting Cyclin A1 expression. The Cyclin A1-CDK2 complex phosphorylates p21 at Thr-57, inducing its cytoplasmic translocation and abrogating its inhibitory effect on cell cycle progression, thereby facilitating G1-S transition. Our findings uncover a critical ALYREF-Cyclin A1-p21 axis in RCC and suggest that targeting this pathway may provide novel therapeutic strategies to overcome pazopanib resistance.

## Introduction

Renal cell carcinoma is a primary malignancy originating from the renal tubular epithelium, accounting for approximately 2% of all cancers^1^. Its global incidence has been steadily increasing, with an estimated 434,840 new cases and 155,953 deaths in 2022^2^. Early-stage RCC is often asymptomatic, and about 33% of patients present with locally advanced or metastatic disease at diagnosis^3^. Advanced RCC is largely unresponsive to conventional radiotherapy and chemotherapy, with a 5-year survival rate below 20%^3^. Targeted therapies have become a mainstay of treatment. Among them, pazopanib, a multitarget receptor tyrosine kinase (RTK) inhibitor, offers effective tumor suppression with relatively mild side effects, making it a first-line option for metastatic RCC^4^. However, most patients develop acquired resistance within two years, leading to disease progression. Elucidating the mechanisms underlying RCC pazopanib resistance is crucial for developing more effective treatments.

Cellular senescence is a stress-responsive cell state marked by stable and typically irreversible cell-cycle arrest^5^, ^6^. Many anticancer treatments, including chemotherapy, radiotherapy, and targeted therapies, can induce senescence known as treatment-induced senescence (TIS)^7^. Senescent cells display phenotypic alterations including enlarged cell morphology with increased cytoplasmic vacuolization, and are commonly characterized by the induction of senescence-associated beta-galactosidase^8^. Senescent cells undergo epigenetic remodeling and secrete a complex array of pro-inflammatory cytokines and extracellular matrix-modifying factors, collectively termed as the senescence-associated secretory phenotype (SASP)^5^, ^9^. In addition, senescent cells are commonly identified based on a combination of molecular features, including loss of proliferative markers, activation of anti-apoptotic^10^ and cell-cycle inhibitory pathways, accumulation of DNA damage markers^9^, formation of nuclear foci enriched in constitutive heterochromatin, and elevated secretion of signaling molecules^5^, ^9^. Although none of these markers is on its own completely specific or universal for all senescence types, there is ample consensus that senescent cells express most of them.

While senescence is widely considered a stable state of cell-cycle arrest, emerging evidence indicates that cancer cells can escape senescence and re-enter the proliferative cycle^9^, ^11^. Tumor cells that exhibit senescent-like phenotypes yet regain proliferative capacity have been reported to contribute to the development of therapeutic resistance^12^-^14^. For example, breast cancer cells acquire resistance to multiple therapies by entering therapy-induced senescence (TIS), and subsequently escape TIS to re-enter the proliferative phase, leading to tumor relapse^15^. Additionally, studies have reported that Acute Myeloid Leukemia (AML) relapse after chemotherapy may be driven by a senescence-like reversion phenotype. Following reversion, these senescent AML cells give rise to relapsed AML with increased stem cell potential^8^. While the association between cellular senescence and drug resistance has been well-documented in various cancers, the relationship between senescence and pazopanib resistance in renal cell carcinoma remains unexplored.

5-methylcytosine (m^5^C) is a crucial post-transcriptional modification on mammalian mRNA^16^, ^17^. Accumulating evidence indicates that m^5^C modification regulates several RNA biological processes, including translation, stability, and export^18^, ^19^. As an m^5^C reader, the export factor Aly/REF (ALYREF) recognizes and binds to m^5^C methylation sites, influencing mRNA splicing, stability, and nucleocytoplasmic transport^19^, ^20^. ALYREF has been implicated in the development of various cancers, including liver, bladder, and cervical cancer. Despite being a crucial component of m^5^C -related genes, the detailed molecular role of ALYREF in RCC pazopanib resistance and its underlying mechanisms remain unclear.

In this study, we found that pazopanib resistant cells exhibited senescence-like phenotype without cell cycle arrest, allowing them to evade apoptosis and remain proliferative under pazopanib treatment. Transcriptome sequencing revealed that ALYREF was highly expressed in resistant cells and tumor models, closely associated with the bypass of cell cycle arrest. Mechanistically, ALYREF specifically recognizes the m^5^C modification on CCNA1, enhancing its mRNA stability and promoting Cyclin A1 expression. Elevated Cyclin A1 binds to CDK2, driving the G1-S transition, while the Cyclin A1-CDK2 complex phosphorylates p21, causing its translocation from the nucleus to the cytoplasm, thereby blocking its inhibitory effect on cell cycle progression and ultimately contributing to pazopanib resistance.

## Results

### Establishment of pazopanib-resistant RCC cell lines and cell derived xenograft models

**Figure 1:**
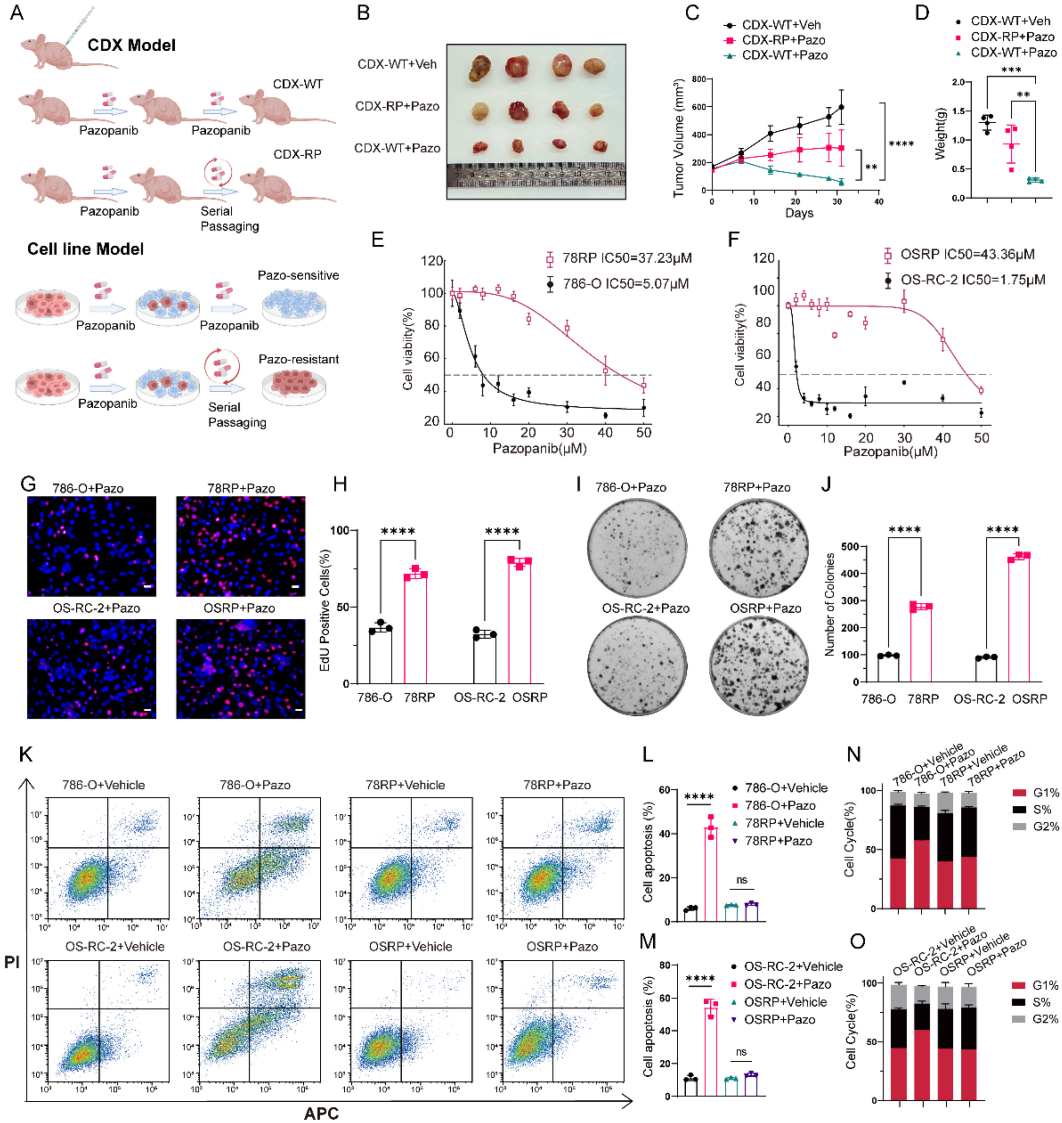
** Establishment of pazopanib resistant RCC cell lines and cell derived xenograft models. (A)** Schematic diagram of establishment of pazopanib-resistant models. **(B-D)** Images (B), volumes (C), and weights (D) of CDX-WT and CDX-RP under pazopanib or vehicle treatment (30 mg/kg, twice a day) for 30 days.** (E, F)** CCK8 assay of Pazopanib-resistant cell lines and control cell lines with pazopanib treatment at indicated concentrations for 48 h. **(G, H)** Representative images of EdU assay and its quantification data of Pazopanib-resistant cell lines and control cell lines with pazopanib treatment. Scale bar, 20μm. **(I, J)** Representative images of colony-formation assay and its quantification data of Pazopanib-resistant cell lines and control cell lines with pazopanib treatment.** (K-M)** Flow cytometric analysis of cell apoptosis and its quantification data of Pazopanib-resistant cell lines and control cell lines with pazopanib treatment.** (N, O)** Flow cytometric analysis of cell cycle in Pazopanib-resistant cell lines and control cell lines with pazopanib treatment. Data are presented as mean ± SD, *P < 0.05, **P < 0.01, ***P < 0.001, ****P < 0.0001; ns, not significant.

To characterize pazopanib resistance in RCC *in vitro* and *in vivo*, we established RCC pazopanib resistant cell lines (78RP and OSRP) and resistant cell derived xenograft (CDX-RP) models building upon the drug resistance induction method established in our previous work^21^** (Figure 1A)**. In the CDX model, daily administration of pazopanib (30 mg/kg, twice a day) via oral gavage was conducted, with tumor size monitored over time. The resulting growth curves and endpoint tumor weights demonstrated that the pazopanib-resistant (CDX-RP) tumors had markedly higher drug tolerance than wild-type (CDX-WT) tumors** (Figure 1, B to D)**. In parallel, CCK-8 assays showed that the IC₅₀ value of resistant cells was markedly higher than that of wild-type cells, reflecting reduced drug sensitivity** (Figure 1, E and F)**. Subsequent EdU and colony formation assays indicated that resistant cells exhibited enhanced colony-formation ability and more active DNA replication compared to wild-type cells under pazopanib treatment **(Figure 1, G to J)**. Furthermore, flow cytometry assays indicated a significantly lower proportion of apoptotic cells in the resistant lines compared to wild-type cells following drug exposure **(Figure 1, K to M)**. Cell cycle profiling showed an increased G1-phase population in wild-type cells following pazopanib treatment, whereas resistant cells showed no significant changes** (Figure 1, N-O and Figure S1, A-B)**. Taken together, these experimental findings indicate that both resistant cells and tumors exhibit greater tolerance to pazopanib compared to their wild-type counterparts, supporting the successful establishment of pazopanib-resistant RCC cell lines and CDX (cell-derived xenograft) models.

### Pazopanib-resistant RCC exhibits senescence-like phenotype contributing to resistance

**Figure 2:**
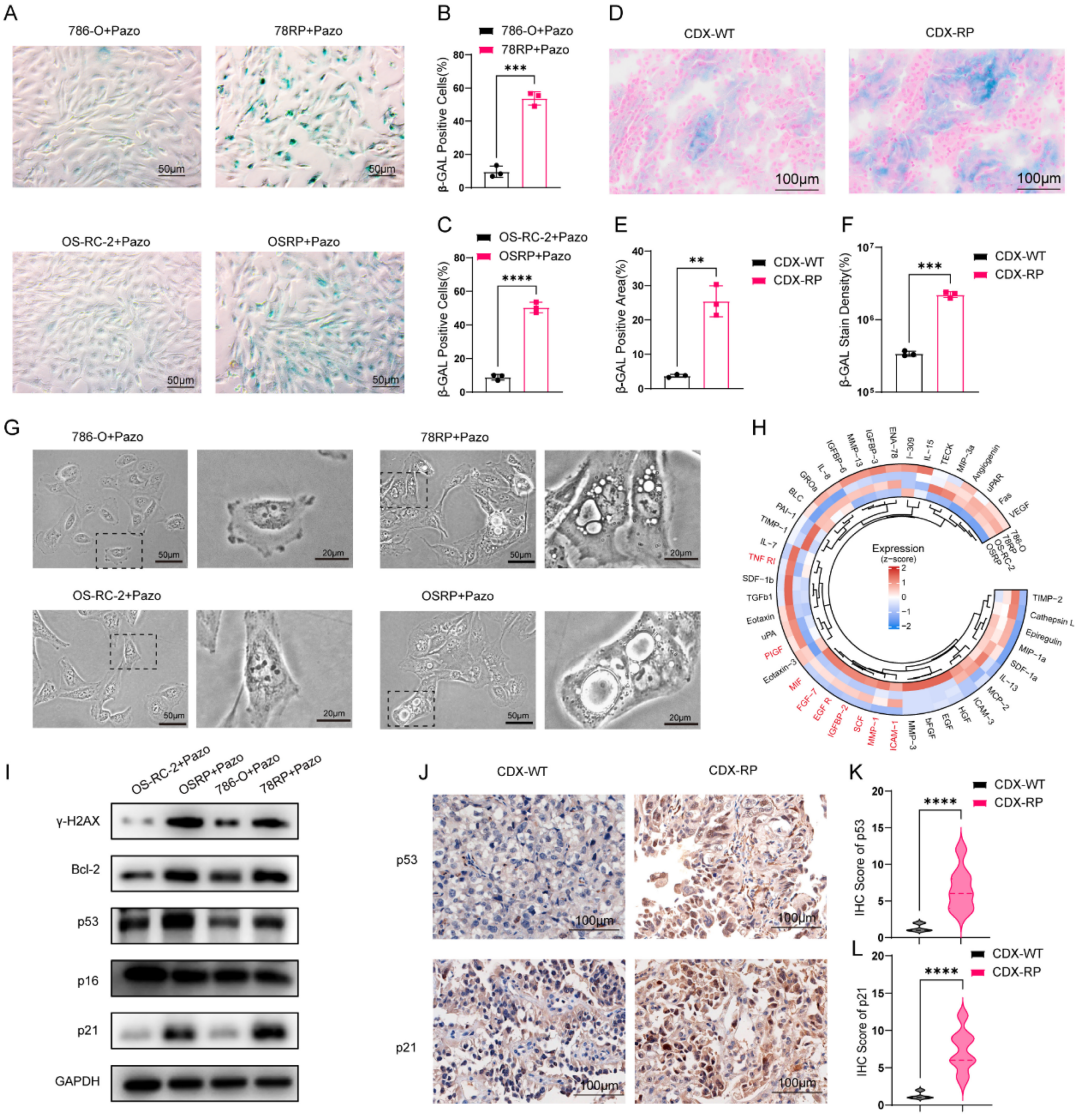
**Pazopanib-resistant RCC exhibits Senescence-like Phenotype. (A-C)** Representative images of β-gal staining assay and its quantification data of pazopanib-resistant cell lines and control cell lines with pazopanib treatment. Scale bar, 50μm.** (D-F)** Representative images of β-gal staining assay of CDX-WT and CDX-RP tumors, accompanied by quantification of staining area and density. Scale bar, 100μm.** (G)** Representative images of cell morphology of pazopanib-resistant cell lines and control cell lines with pazopanib treatment. **(H)** Circos plot showing differential expression of SASP-associated cytokines in wild-type (786-O, OS-RC-2) and pazopanib-resistant (78RP, OSRP) renal cancer cells.** (I)** Western blotting of the γ-H2AX, Bcl-2, p53, p21, p16 in pazopanib-resistant cell lines and control cell lines with pazopanib treatment. **(J-L)** Representative IHC staining images for p53 and p21 protein in CDX-WT and CDX-RP tumors are presented. Scale bar, 100μm. IHC scores are calculated and analyzed. Data are presented as mean ± SD, *P < 0.05, **P < 0.01, ***P < 0.001, ****P < 0.0001; ns, not significant.

To explore the underlying relevance between cell senescence and pazopanib resistance, we conducted β-gal staining to detected senescent cells. The analysis revealed a significantly higher number of positively stained cells in 78RP and OSRP population relative to 786-O and OS-RC-2 **(Figure 2, A-C and Figure S2, A-B)**. Consistent findings were observed in frozen sections of tumor tissues, where CDX-RP tumor group exhibited increased staining intensity and positively stained area than CDX-WT group **(Figure 2, D to F)**. Recognizing that β-gal staining is insufficient to conclusively define cellular senescence, we further examined whether resistant cells display other hallmarks of the senescent phenotype. Under pazopanib treatment, both 78RP and OSRP cells exhibited senescence-like morphology, including increased cytoplasmic vacuolization and cellular flattening **(Figure 2, G)**. We further collected the culture supernatants from wild-type and resistant cells after 48 hours of pazopanib treatment and performed cytokine array analysis. The analysis revealed a notable enrichment of SASP-associated cytokines in the conditioned medium derived from resistant cells **(Figure 2, H and Figure S3, A-B)**. In addition, results of Western blot and IHC indicated the elevated levels of p53 and p21 in pazo-resistant group compared to wild-type counterparts** (Figure 2, I- L and Figure S4, A)**. Notably, increased expression of γ-H2AX, a DNA damage marker, and Bcl-2, an anti-apoptotic factor, was also observed in the resistant cells **(Figure 2, I)**. Collectively, these findings indicate that pazopanib-resistant cells display senescence-like phenotype upon pazopanib exposure.

**Figure 3:**
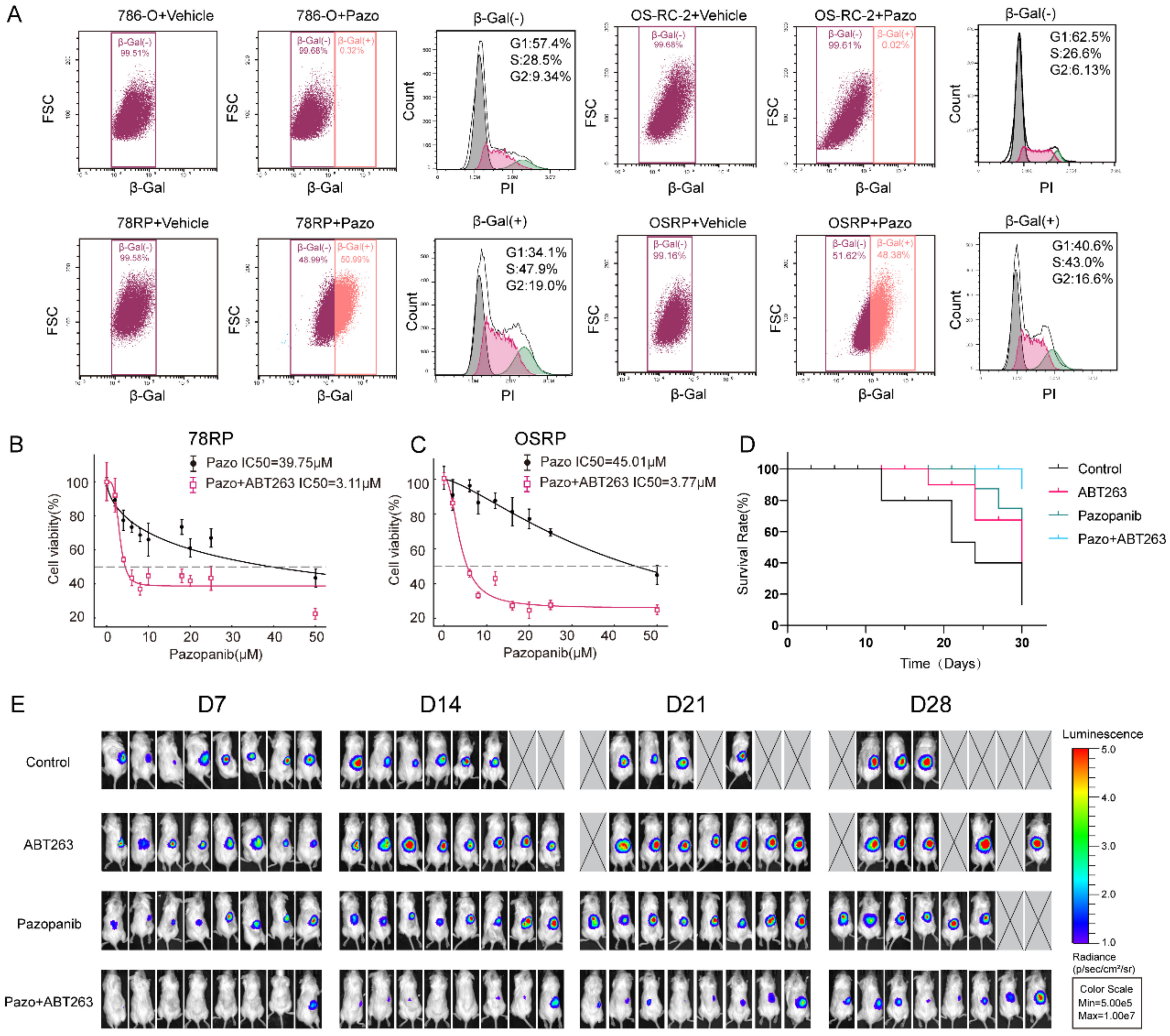
**Senescence-like Phenotype Contributes to resistance. (A)**Flow cytometry analysis of SA-β-galactosidase activity using the CellEvent™ Senescence Green Probe Kit in wild-type (786-O, OS-RC-2) and resistant (78RP, OSRP) renal cancer cells treated with vehicle or pazopanib. β-Gal (+) and β-Gal (-) populations were gated based on staining intensity. Sorted β-Gal (+) and β-Gal (-) populations were further analyzed for cell cycle using propidium iodide (PI) staining.** (B, C)** Dose-response curves of resistant cells treated with increasing concentrations of pazopanib alone or in combination with 100 nM ABT263 for 48 hours. **(D)** Kaplan-Meier survival curves of mice bearing orthotopic OSRP-derived renal tumors treated with vehicle (control), ABT263(100mg/kg, once a day), pazopanib (30mg/kg, twice a day), or the combination of ABT263 and pazopanib. **(E)** Representative bioluminescence imaging of orthotopic renal tumor-bearing mice treated with vehicle, ABT263(100mg/kg, once a day), pazopanib (30mg/kg, twice a day), or the combination of ABT263 and pazopanib. Data are presented as mean ± SD, *P < 0.05, **P < 0.01, ***P < 0.001, ****P < 0.0001; ns, not significant.

Unlike typical cellular senescence, our experimental results revealed that pazopanib resistant cells exhibited senescence-like phenotype without undergoing cell cycle arrest. To further validate this observation, we performed β-galactosidase staining using the CellEvent™ Senescence Green Probe Kit and analyzed the cells via flow cytometry. Cells treated with vehicle were used as a baseline and defined as β-Gal(-), while those with higher β-Gal staining intensity were classified as β-Gal(+). Flow cytometry was used to isolate β-Gal (+) populations from both wild-type and resistant cells for cell cycle analysis. A substantial number of β-Gal (+) cells was detected in the resistant group which showed no evidence of G1-S phase arrest. In contrast, pazopanib treatment had minimal impact on β-Gal staining intensity in wild-type cells** (Figure 3, A)**. These findings further support our conclusion that resistant cells display a senescence-like phenotype in the absence of cell cycle arrest.

Given that these cells exhibited marked resistance to apoptosis under pazopanib treatment and showed elevated expression of the anti-apoptotic protein Bcl-2, we next tested the efficacy of combining pazopanib with ABT-263—a Bcl-2 family inhibitor commonly used in senolytic strategies—to evaluate its cytotoxic effect on resistant cells. We first determined the IC₅₀ values of ABT-263 using CCK-8 assays, which were 1325 nM for 78RP and 1974 nM for OSRP cells, respectively** (Figure S5, A)**. Notably, the concentration of 100 nM ABT-263, which is commonly used in cell-based studies according to the literature^22^, ^23^, was found to be substantially lower than measured IC₅₀ in our resistant cell lines. Time-course CCK-8 assays further confirmed that 100 nM ABT-263 alone exerted minimal effect on cell viability** (Figure S5, B and C)**. However, when combined with pazopanib, co-treatment with 100 nM ABT-263 significantly enhanced the sensitivity of resistant cells to pazopanib, as evidenced by a marked reduction in the IC₅₀ **(Figure 3, B and C)**. Consistent with *in vitro* findings, *in vivo* experiments demonstrated that the combination of ABT-263 and pazopanib significantly suppressed tumor growth in a murine orthotopic model and improved overall survival at the 30-day endpoint **(Figure 3, D-E and Figure S6, A-B)**. Collectively, these results support the notion that co-treatment with the senolytic agent ABT263 led to a notable reduction in pazopanib resistance. This finding further highlights the functional contribution of these senescence-like cells lacking cell cycle arrest in promoting resistance to pazopanib.

### ALYREF drives pazopanib resistance through cell-cycle regulation

**Figure 4:**
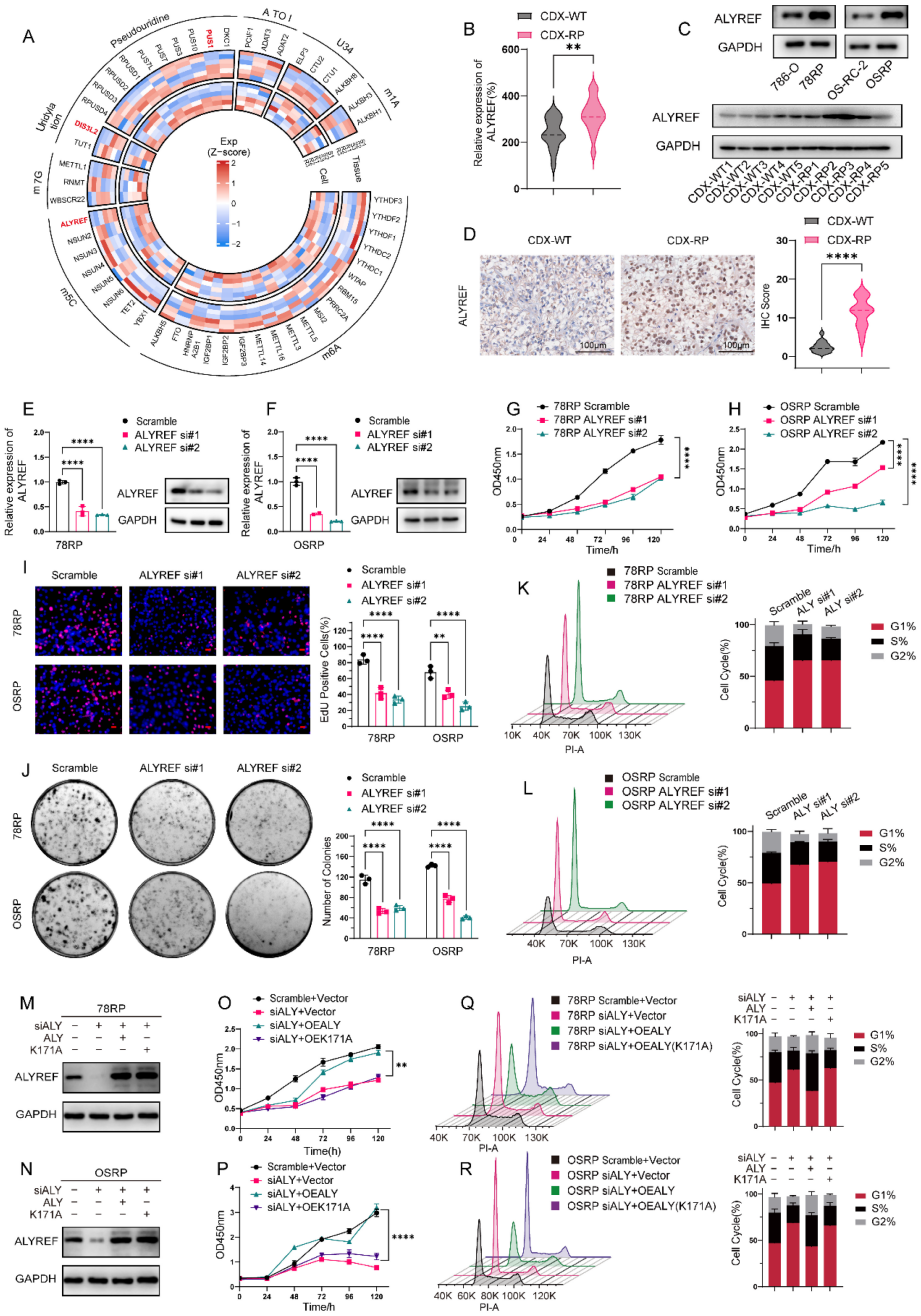
**ALYREF Drives Pazopanib Resistance Through Cell-Cycle Regulation (A)** Transcriptome profiling of RNA modification-related genes in pazopanib-resistant cells and tumors. **(B)** RT-qPCR analysis of ALYREF in CDX-WT and CDX-RP tumor tissues. **(C)** Western blot analysis of ALYREF protein expression in pazopanib-resistant cell lines (78RP and OSRP) and their wild-type counterparts (786-O and OS-RC-2) (top panel), as well as in cell-derived xenograft tumors (CDX-RP and CDX-WT, bottom panel). **(D)** Representative IHC staining images for ALYREF protein in CDX-WT and CDX-RP tumors are presented. IHC scores are calculated and analyzed. Scale bar, 100μm. **(E, F)** RT-qPCR and Western blot analysis of ALYREF expression following siRNA-mediated knockdown in 78RP (left) and OSRP (right) cells.** (G, H)** CCK8 assay of Pazopanib-resistant cell lines (78RP and OSRP) following ALYREF knockdown using two independent siRNAs, measured at the indicated time points.** (I)** Representative images of EdU assay and its quantification data of Pazopanib-resistant cell lines (78RP and OSRP) following ALYREF knockdown. Scale bar, 20μm. **(J)** Representative images of colony-formation assay and its quantification data of Pazopanib-resistant cell lines (78RP and OSRP) following ALYREF knockdown.** (K, L)** Flow cytometric analysis of cell cycle and its quantification data of Pazopanib-resistant cell lines (78RP and OSRP) following ALYREF knockdown.** (M, N)** Western blot analysis of ALYREF knockdown and ectopic expression of either wild-type ALYREF or the m^5^C-binding-deficient mutant (K171A) in 78RP (M) and OSRP (N) cells.** (O, P)** CCK8 assay of Pazopanib-resistant cell lines (78RP and OSRP) following ALYREF knockdown and rescue with either wild-type ALYREF or m^5^C-binding-deficient mutant (K171A), measured at the indicated time points. **(Q, R)** Flow cytometric analysis of cell cycle and its quantification data of Pazopanib-resistant cell lines (78RP and OSRP) following ALYREF knockdown and rescue with either wild-type ALYREF or m^5^C-binding-deficient mutant (K171A). Data are presented as mean ± SD, *P < 0.05, **P < 0.01, ***P < 0.001, ****P < 0.0001; ns, not significant.

In light of the contribution of this phenotype to drug resistance, we aimed to elucidate its underlying mechanisms. RNA was extracted from 78RP and 786-O cells, as well as from CDX-R and CDX-WT tumors, and subjected to transcriptome sequencing. In our previous work, we identified a close relationship between m6A modification and sunitinib resistance in renal cell carcinoma, recognizing that epigenetic alterations—particularly RNA modifications—play a key role in the development of acquired resistance^16^. Consequently, our analysis focused on RNA modification related genes within the above sequencing datasets^24^, ^25^. Sequencing results revealed elevated expression of ALYREF, PUS1, and DIS3L2 in both resistant cells and CDX-RP tissues **(Figure 4, A and Supplementary Table S1-2)**. We further filtered the sequencing data using a threshold of absolute log₂(fold change) ≥ 1, and identified ALYREF as the only candidate gene that was consistently and significantly upregulated in both resistant cells and resistant tumor tissues** (Figure S7, A to F)**. Western blot and immunohistochemical analyses further confirmed elevated ALYREF protein levels in the resistant group** (Figure 4, C and D)**. Consistently, clinical data analysis from public databases and our internal cohort revealed that high ALYREF expression is significantly associated with poor prognosis in RCC patients, highlighting its clinical relevance **(Figure S8, A and B)**. To investigate the physiological role of ALYREF in resistant cells, we knocked down ALYREF in 78RP and OSRP with siRNAs **(Figure 4, E and F)**. Flow cytometric analysis revealed that ALYREF knockdown markedly increased the proportion of cells in the G1 phase, indicating pronounced G1 phase arrest **(Figure 4, K and L)**. Subsequent CCK-8, colony formation, and EdU assays consistently demonstrated that ALYREF knockdown significantly increased the sensitivity of resistant cells to pazopanib** (Figure 4, G-J and Figure S9, A-B)**.

To assess whether ALYREF's role in pazopanib-resistant cells relies on its m^5^C reader function, we generated a recognition site mutant (K171A) plasmid of ALYREF^20^. In ALYREF-knockdown resistant cells, ectopic expression of wild-type ALYREF overexpression reduced G1-phase arrest and restored pazopanib resistance, whereas the mutant ALYREF had no effect on the G1-phase and failed to restore drug resistance **(Figure 4, M-R and Figure S9, E-F)**. These results substantiated the importance of ALYREF's m^5^C reader activity in regulating the cell cycle of resistant cells. We also overexpressed wild-type and mutant ALYREF(K171A) in 786-O and OS-RC-2 cells **(Figure S10, A and B)**. Consistent with the results in resistant cells, wild-type ALYREF reduced G1-phase arrest and enhanced pazopanib resistance, while the mutant ALYREF(K171A) had no significant effect on the cell cycle or drug resistance **(Figure S10, C to H)**.

Given our findings, we turned to *in vivo* models to probe ALYREF's role in pazopanib resistance. We performed orthotopically injection of luciferase labeled OSRP cell in nude mice, revealing that ALYREF silencing significantly impaired pazopanib resistance **(Figure S10, I to K)**. Taken together, these findings demonstrate that ALYREF, as an m^5^C modification reader, drives pazopanib resistance through cell cycle regulation.

### CCNA1 functions as a key downstream effector of ALYREF in mediating pazopanib resistance

**Figure 5:**
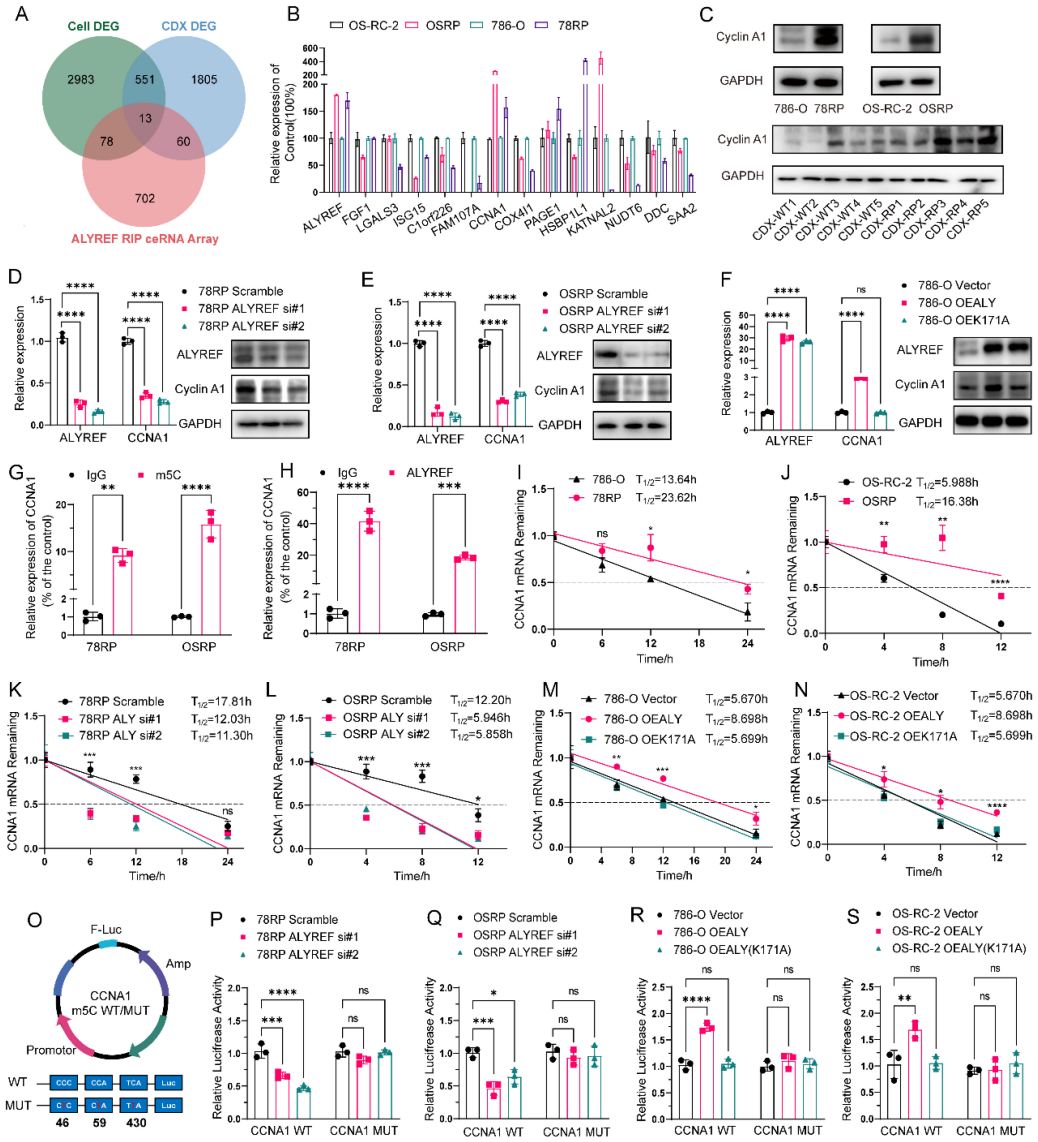
**ALYREF enhances CCNA1 expression by recognizing m^5^C-modified mRNA. (A)** Venn diagram showing overlap of differentially expressed genes (DEGs) from resistant cells, resistant CDX tumors, and ALYREF RIP ceRNA array results. 13 candidate target genes were identified at the intersection.** (B)** qPCR validation of the 13 candidate target genes in pazopanib-resistant cell lines (78RP and OSRP) and their wild-type counterparts (786-O and OS-RC-2). **(C)** Western blot analysis of Cyclin A1 protein expression in pazopanib-resistant cell lines (78RP and OSRP) and their wild-type counterparts (786-O and OS-RC-2) (top panel), as well as in cell-derived xenograft tumors (CDX-RP and CDX-WT, bottom panel).** (D, E)** qPCR and Western blot analysis of ALYREF and CCNA1 expression in pazopanib-resistant renal cancer cells following ALYREF knockdown.** (F)** qPCR and Western blot analysis of ALYREF and CCNA1 expression in 786-O cells overexpressing wild-type or mutant ALYREF. **(G, H)** RNA immunoprecipitation (RIP) analysis of CCNA1 mRNA enrichment by anti- m^5^C and anti-ALYREF antibodies in resistant cell lines.** (I, J)** Analysis of CCNA1 mRNA half-life in wild-type and resistant renal cancer cell lines.** (K, L)** Analysis of CCNA1 mRNA half-life following ALYREF knockdown in resistant renal cancer cell lines.** (M, N)** Analysis of CCNA1 mRNA half-life following ALYREF overexpression in wild-type RCC.** (O)** Schematic diagram of luciferase reporter constructs containing wild-type or m^5^C site-mutated (C-to-T mutation, 46, 59 and 430 sites) CCNA1 mRNA sequences. **(P, Q)** Relative luciferase activity of CCNA1 mRNA constructs containing wild-type or mutant seed sequences after co-transfection with ALYREF siRNAs or scramble control. **(R, S)** Relative luciferase activity of CCNA1 mRNA constructs containing wild-type or mutant seed sequences after co-transfection with wild-type or mutant ALYREF. Data are presented as mean ± SD, *P < 0.05, **P < 0.01, ***P < 0.001, ****P < 0.0001; ns, not significant.

To identify the target genes of ALYREF, RNA immunoprecipitation (RIP) was performed in 78RP cells using anti-ALYREF antibody, and the RIP products were analyzed with Human ceRNA Microarray. Candidate genes were ranked by fold change and p-value (p <0.05 and fold change ≥ 2), and the top hits were intersected with differentially expressed genes from transcriptome data comparing resistant and wild-type RCC models in both cell lines and xenograft tumors, yielding 13 potential targets:* FGF1, LGALS3, CCNA1, ISG15, C1orf226, FAM107A, COX4I1, PAGE1, HSBP1L1, KATNAL2, NUDT6, DDC,* and* SAA2* **(Figure 5, A)**. Validation of these genes in 78RP and OSRP cells revealed that only CCNA1, which encodes cyclin A1, was consistently and markedly upregulated in pazopanib resistant cell lines **(Figure 5, B)**. Western blot analysis further confirmed elevated Cyclin A1 protein expression in resistant cells and tumor tissue **(Figure 5, C)**. Cyclin A1 is an alternative CDK2 associated A-type cyclin primarily expressed in the testis, involved in meiosis and spermatogenesis^26^, ^27^. However, Aberrant overexpression of Cyclin A1 has been reported in AML and other malignancies^28^, ^29^. Notably, survival analysis using both public databases and our internal cohort demonstrated that high CCNA1 expression is significantly associated with poor prognosis in RCC patients, further supporting its clinical significance **(Figure S11 A and B)**. In parallel, functional studies have shown that Cyclin A1/CDK2 complex can drive G1-S phase progression in both somatic cells and leukemic blasts^28^, ^30^.

To investigate the intrinsic relationship between ALYREF and CCNA1, we knocked down and overexpressed ALYREF using siRNA and plasmids. qPCR and Western blot analyses showed that ALYREF knockdown resulted in decreased mRNA and protein levels of CCNA1, while overexpression of wild-type ALYREF but not recognition site mutant (K171A) resulted in an elevation of CCNA1 mRNA and protein levels **(Figure 5, D-F and Figure S12, A)**. These results suggested a positive regulatory relationship between ALYREF and CCNA1, potentially linked to m^5^C modification. Consistently, correlation analysis revealed a significant positive association between ALYREF and CCNA1 expression in both the TCGA-KIRC cohort and our internal SRRSH cohort, providing clinical support for the proposed regulatory axis in RCC patients** (Figure S13, A and B)**. Further RIP experiments using anti- m^5^C and anti-ALYREF antibodies revealed that the CCNA1 mRNA content in the RIP products was significantly higher than in the IgG control, indicating the presence of m^5^C modification on CCNA1 mRNA and that ALYREF specifically recognize CCNA1 mRNA **(Figure 5, G and H)**.

Subsequently, we sought to explore the effect of m^5^C modification on CCNA1. Given that ALYREF positively regulates CCNA1 mRNA levels, we aimed to investigate its impact on the stability of CCNA1 mRNA. RNA stability assays showed that in 78RP and OSRP cells, the half-life of CCNA1 mRNA was significantly longer compared to 786-O and OS-RC-2** (Figure 5, I and J)**. Knockdown of ALYREF notably reduced the mRNA half-life, while overexpression of wild-type ALYREF—but not the K171A binding site mutant—significantly prolonged the half-life of CCNA1 mRNA **(Figure 5, K to N)**. To further substantiate this assertion, we conducted online prediction by m^5^C Finder to identify potential m^5^C modification sites on CCNA1 mRNA. Based on these predictions, we constructed both WT (CCNA1 WT) and m^5^C sites mutated CCNA1 mRNA (CCNA1 MUT) luciferase reporter plasmids **(Figure 5, O)**. The dual luciferase reporter assay revealed that relative luciferase activity diminished in the CCNA1 WT group when ALYREF was knocked down, while no significant change was observed in the m^5^C sites mutant (CCNA1 MUT) group **(Figure 5, P and Q)**. Overexpression of ALYREF resulted in increased luciferase activity in the CCNA1 WT group, whereas overexpression of ALYREF (K171A) failed to induce a significant increase **(Figure 5, R and S)**. Similarly, in the CCNA1 MUT group, neither ALYREF nor ALYREF (K171A) affected luciferase activity **(Figure 5, R and S)**. Collectively, these findings indicate that ALYREF recognizes the m^5^C modification on CCNA1 mRNA and promotes its stability, leading to upregulated expression.

**Figure 6:**
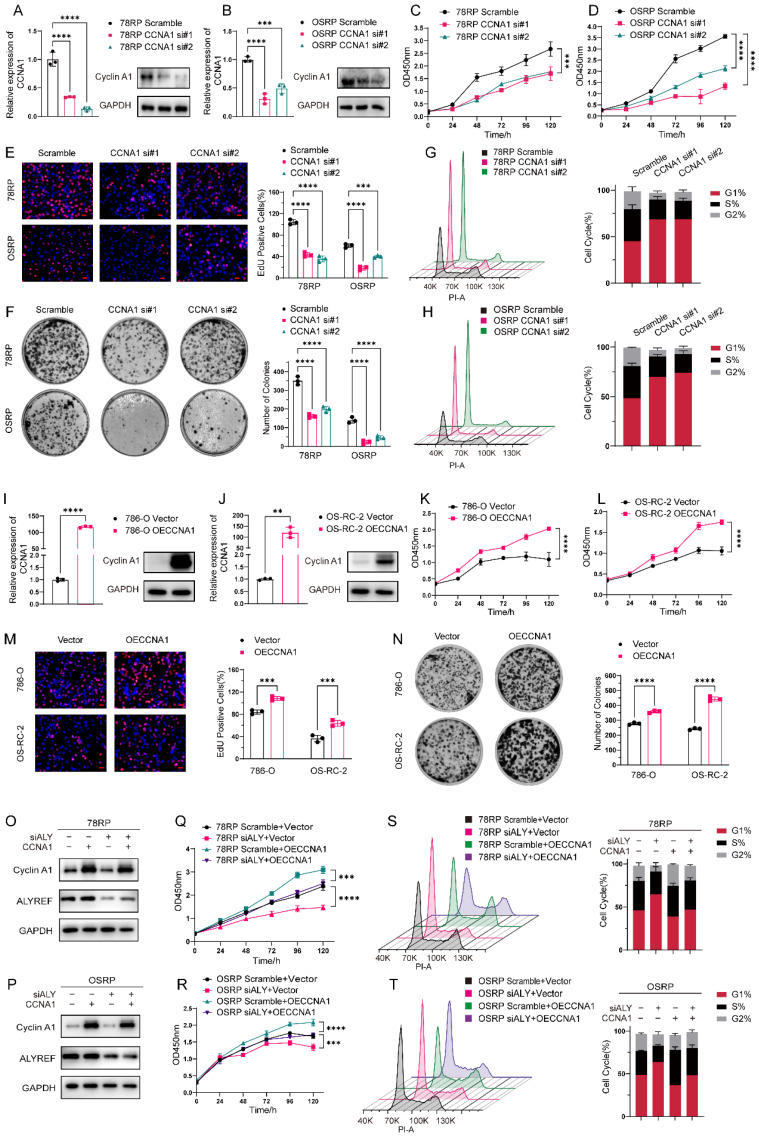
**CCNA1 Functions as a Key Downstream Effector of ALYREF in Mediating Pazopanib Resistance. (A, B)** RT-qPCR and Western blot analysis of CCNA1 expression following siRNA-mediated knockdown in 78RP (left) and OSRP (right) cells. **(C, D)** CCK8 assay of Pazopanib-resistant cell lines (78RP and OSRP) following CCNA1 knockdown using two independent siRNAs, measured at the indicated time points.** (E)** Representative images of EdU assay and its quantification data of Pazopanib-resistant cell lines (78RP and OSRP) following CCNA1 knockdown. Scale bar, 20μm. **(F)** Representative images of colony-formation assay and its quantification data of Pazopanib-resistant cell lines (78RP and OSRP) following CCNA1 knockdown.** (G, H)** Flow cytometric analysis of cell cycle and its quantification data of Pazopanib-resistant cell lines (78RP and OSRP) following CCNA1 knockdown.** (I, J)** RT-qPCR and Western blot analysis of CCNA1 expression following ectopic expression of CCNA1 in 786-O (left) and OS-RC-2 (right) cells.** (K, L)** CCK8 assay of RCC cell lines (786-O and OS-RC-2) following ectopic expression of CCNA1, measured at the indicated time points.** (M)** Representative images of EdU assay and its quantification data of 786-O and OS-RC-2 following overexpression of CCNA1. Scale bar, 20μm. **(N)** Representative images of colony-formation assay and its quantification data of 786-O and OS-RC-2 following overexpression of CCNA1. **(O, P)** Western blot analysis of ALYREF knockdown and ectopic expression of CCNA1 in 78RP (M) and OSRP (N) cells. **(Q, R)** CCK8 assay assessing the functional rescue effect of CCNA1 overexpression upon ALYREF knockdown in 78RP (Q) and OSRP (R) cells, measured at the indicated time points.** (S, T)** Flow cytometric analysis of cell cycle and its quantification data of 78RP(S) and OSRP(T) following ALYREF knockdown and rescue with CCNA1 overexpression. Data are presented as mean ± SD, *P < 0.05, **P < 0.01, ***P < 0.001, ****P < 0.0001; ns, not significant.

Having identified CCNA1 as a key target of ALYREF, we further set to explore its contribution to ALYREF-mediated pazopanib resistance. Flow cytometric analysis revealed that CCNA1 knockdown markedly increased the proportion of cells in the G1 phase, indicating pronounced G1 phase arrest **(Figure 6, G and H)**. Conversely, overexpression of CCNA1 reduced the G1-phase cell population, suggesting its role in promoting cell cycle progression** (Figure S14, A)**. Subsequent CCK-8, colony formation, and EdU assays consistently demonstrated that CCNA1 knockdown significantly increased the sensitivity of resistant cells to pazopanib **(Figure 6, C-F and Figure S9 G-H)**, with the opposite effect observed upon CCNA1 overexpression** (Figure 6, K- N and Figure S9 I-J)**. Rescue experiments further demonstrated that CCNA1 overexpression could reverse the effects of ALYREF knockdown, alleviating G1-phase arrest and restoring pazopanib resistance **(Figure 6, O-T and Figure S9 K-L)**. Taken together, these findings demonstrate that CCNA1 functions as a critical downstream target of ALYREF, mediating its role in cell cycle regulation and the promotion of pazopanib resistance.

### Cyclin A1-CDK2 mediated phosphorylation drives cytoplasmic translocation of p21

**Figure 7:**
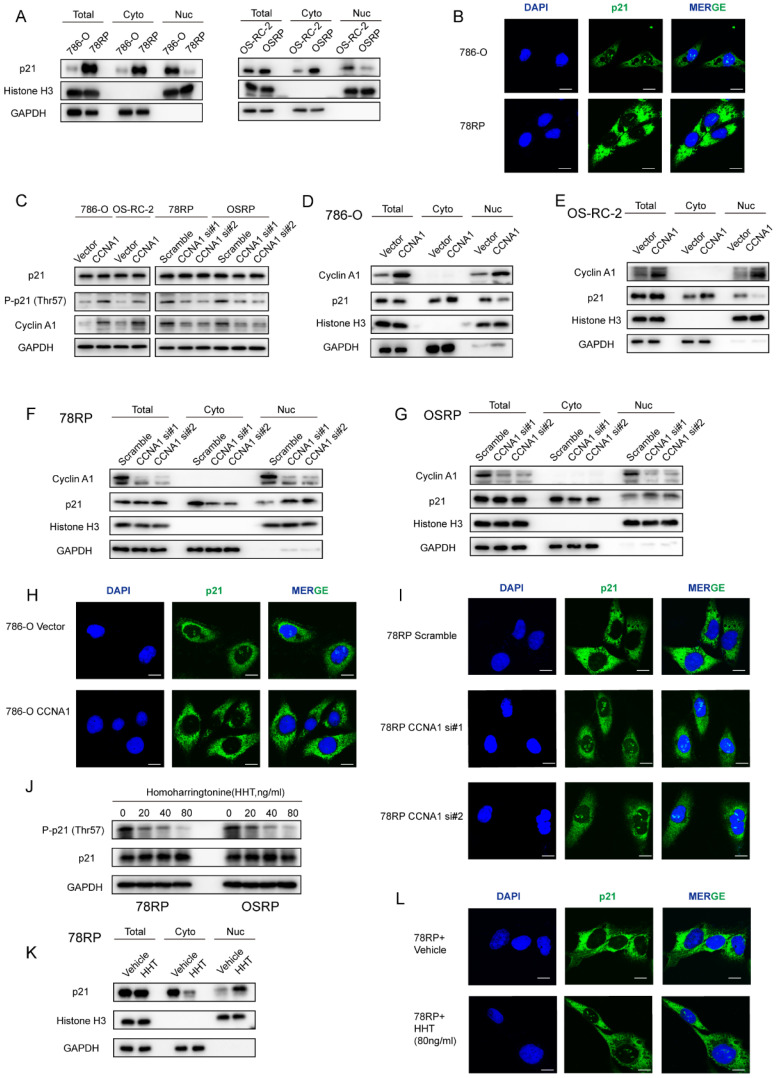
**Cyclin A1-CDK2 Mediated Phosphorylation Drives Cytoplasmic Translocation of p21. (A)** Western blot analysis of p21 subcellular distribution in wild-type and pazopanib-resistant renal cancer cells. **(B)** Immunofluorescence staining of p21 in wild-type and pazopanib-resistant renal cancer cells. Representative images of 786-O and 78RP cells stained with anti-p21 antibody (green) and DAPI (blue). Scale bar, 20μm.** (C)** Western blot analysis of total and phosphorylated p21 (Thr-57) in renal cancer cells following CCNA1 overexpression or knockdown.** (D, E)** Western blot analysis of p21 subcellular distribution in 786-O(D) and OS-RC-2(E) following CCNA1 overexpression. **(F, G)** Western blot analysis of p21 subcellular distribution in 78RP(F) and OSRP(G) following CCNA1 knockdown.** (H)** Immunofluorescence staining of p21 in 786-O following CCNA1 overexpression. Scale bar, 20μm. **(I)** Immunofluorescence staining of p21 in 78RP following CCNA1 knockdown. Scale bar, 20μm. **(J)** Western blot analysis of p21 phosphorylation in 78RP and OSRP cells treated with increasing concentrations of HHT (0-80 ng/ml). **(K)** Western blot analysis of p21 subcellular distribution in 78RP cells following HHT treatment.** (L)** Immunofluorescence staining of p21 in 78RP cells following HHT treatment. Scale bar, 20μm.

Accumulation of p21 is critical for maintaining cell-cycle arrest in senescent cells^31^. Despite robust p21 expression, resistant cells failed to undergo cell-cycle arrest, suggesting that p21 may function differently in this setting. Studies have shown that the physiological functions of p21 are closely associated with its subcellular localization^32^. Nuclear p21 primarily acts as a tumor suppressor, whereas cytoplasmic p21 exhibits oncogenic behavior by inhibiting apoptosis and promoting tumor progression^31^, ^33^. To explore its functional role, we first assessed the subcellular distribution of p21. Unexpectedly, cytoplasmic-nuclear fractionation and immunofluorescence assays revealed that p21 was predominantly localized in the cytoplasm rather than the nucleus in resistant cells** (Figure 7, A and B)**. Among the factors influencing p21 localization, phosphorylation is a common determinant^34^-^36^. Considering that the CyclinA1-CDK2 complex can bind to p21 and induce phosphorylation at Thr-57^37^, we aimed to explore whether the cytoplasmic localization of p21 in resistant cells is associated with CyclinA1-CDK2-mediated phosphorylation. Western blot analysis showed that overexpression of CCNA1 in 786-O and OS-RC-2 significantly increased the Thr-57 phosphorylation of p21, whereas CCNA1 knockdown in resistant cells led to a reduction in Thr-57 phosphorylation **(Figure 7, C)**. Further cytoplasmic-nuclear fractionation and immunofluorescence assays revealed that CCNA1 overexpression promoted cytoplasmic accumulation of p21 and reduced its nuclear localization, with the opposite effect observed upon CCNA1 knockdown **(Figure 7, D to I)**. To further confirm the role of the CyclinA1-CDK2 complex, we treated resistant cells with homoharringtonine (HHT), a compound that specifically disrupts CyclinA1-CDK2 binding^38^. Western blot analysis revealed that HHT treatment inhibited phosphorylation of p21 at Thr-57 in a dose-dependent manner **(Figure 7, J)**. Furthermore, cytoplasmic-nuclear fractionation and immunofluorescence assays demonstrated that HHT promoted nuclear accumulation of p21 while reducing its cytoplasmic localization** (Figure 7, K and L)**. These findings indicate that CyclinA1-CDK2-mediated phosphorylation at Thr-57 promotes the cytoplasmic translocation of p21.

## Discussion

Metastatic RCC (mRCC) shows limited response to chemotherapy and radiotherapy, with a 5-year survival rate of only 12%^3^. Targeted therapy, alone or in combination with immunotherapy, remains the current standard of care. Pazopanib is a first-line VEGFR tyrosine kinase inhibitor (TKI) that blocks VEGF and PDGF pathways, effectively suppressing tumor angiogenesis. Compared to sunitinib, another first-line TKI, pazopanib has lower affinity for Flt-3 and reduced hematologic toxicity^39^. The COMPARZ trial demonstrated non-inferior progression-free survival (PFS) between pazopanib and sunitinib (8.4 vs. 9.5 months), with a higher objective response rate (ORR) in the pazopanib group (31% vs. 25%, P = 0.03)^40^. Furthermore, patients treated with pazopanib experienced fewer severe adverse events, supporting its superior safety and tolerability profile^40^. Despite initial efficacy, most patients develop acquired resistance to pazopanib within two years, leading to recurrence or progression. This acquired resistance remains a major challenge in mRCC management and significantly limits long-term outcomes. Therefore, elucidating the mechanisms underlying pazopanib resistance is critical for improving the prognosis of patients with metastatic RCC.

Cellular senescence is a stress-induced state characterized by stable and typically irreversible cell-cycle arrest. In cancer therapy, prolonged drug exposure frequently induces such stress responses in tumor cells. These senescent tumor cells exhibit intrinsic resistance to apoptosis due to elevated expression of anti-apoptotic Bcl-2 family proteins, allowing survival and accumulation during treatment^10^. Although traditionally considered irreversible, senescence escape has been increasingly observed in cancer, contributing to therapeutic resistance and relapse^41^. Our study revealed that pazopanib-resistant cells display pronounced senescence-like phenotypes while bypassing cell-cycle arrest, diverging from classical senescence, and instead resembling cells that have escaped from senescence. Previous work in acute leukemia has shown that chemotherapy can induce reversible senescence, and tumor cells that recover from the senescent state retain senescence-like phenotypes while exhibiting enhanced clonogenic potential and tumor-regrowth capacity^8^. However, in most studies investigating therapy resistance driven by senescence escape, the senescence-like phenotype is transient and serves only as a temporary adaptive state, disappearing as tumor cells repopulate^13^, ^15^. By contrast, in our model, resistant cells maintained senescence-like phenotypes over time. We propose that this persistence may be related to the duration and manner of drug exposure. Unlike prior studies that induce senescence followed by treatment withdrawal, we gradually escalated drug concentrations over time, applying continuous pharmacologic pressure. This may contribute to the stable senescence-like phenotype observed. Moreover, while previous studies have monitored the transition from growth arrest to proliferation, our analysis was limited to the start and end points of resistance, missing intermediate cellular changes, which remains a limitation of this study and warrants further investigation.

We propose that this unique form of senescence is driven by the ALYREF-CCNA1 axis. The Cyclin A family governs cell-cycle progression by forming complexes with cyclin-dependent kinases (CDKs). The activities of CDK2-Cyclin A and CDK1-Cyclin A are essential for S-phase entry and mitotic progression, respectively. The Cyclin A family comprises two isoforms in humans: Cyclin A1 and Cyclin A2. Unlike Cyclin A2, which is broadly expressed during the S and G2/M phases of the somatic cell cycle, Cyclin A1 is primarily expressed in the testis, involved in meiosis and spermatogenesis^26^. However, Aberrant overexpression of Cyclin A1 has been reported in AML and other malignancies^42^, ^43^. In parallel, functional studies have shown that Cyclin A1 can drive G1-S phase progression in both somatic cells and leukemic blasts^30^. Consistent with these findings, our study demonstrates that Cyclin A1 is markedly upregulated in pazopanib-resistant cells and facilitates G1-S phase transition.

High expression of the CDK2 inhibitor p21^Cip1/Waf1^ (CDKN1A) and the CDK4/6 inhibitor p16^INK4a^ (CDKN2A) is critical for maintaining cell-cycle arrest in senescent cells. Accumulation of p21 and p16 leads to sustained activation of the RB protein family, which in turn represses E2F transcription factors and inhibits the expression of genes required for cell-cycle progression^44^. However, unlike most cell-cycle-regulated genes, the CCNA1 promoter is not controlled by E2F transcription factors^45^. This transcriptional independence may explain the persistent overexpression of CCNA1 in senescent-like drug-resistant cells. In our study, resistant cells exhibited high levels of p53 and p21, while p16 expression remained comparable to that of parental cells. Despite robust p21 expression, resistant cells failed to undergo cell-cycle arrest, suggesting that p21 may function differently in this setting. p21 is a pleiotropic protein involved in numerous cellular processes, including cell-cycle regulation, apoptosis, differentiation, migration, cytoskeletal dynamics, transcription, DNA repair, and both the initiation and maintenance of senescence. Notably, its biological effects are closely linked to subcellular localization: nuclear p21 primarily enforces cell-cycle arrest and tumor suppression, whereas cytoplasmic p21 can act as an oncogene by inhibiting apoptosis and promoting tumor progression. Consistent with this duality, we found that resistant cells escape cell-cycle arrest through Cyclin A1-CDK2-mediated phosphorylation, which drives cytoplasmic translocation of p21 and disables its cell-cycle inhibitory function. Our findings uncover a novel mechanism by which senescent-like cells evade cell-cycle arrest and offer new therapeutic insights into overcoming pazopanib resistance in renal cell carcinoma.

Therapy-induced senescence (TIS) and its contribution to acquired resistance have been documented across various tyrosine kinase inhibitors (TKIs), such as the EGFR/HER2 inhibitor lapatinib in breast cancer and the BCR-ABL inhibitor imatinib in leukemia^46^, ^47^. Recognizing TIS as a vulnerable transitional state rather than a permanent endpoint highlights a critical therapeutic window. Consequently, the clinical potential of combining TKI therapy with senolytic agents warrants particular attention. In preclinical breast cancer and leukemia models, applying Navitoclax (ABT-263) following TKI treatment successfully eradicated senescent tumor cells, mitigated pro-survival signaling, and delayed recurrence^48^. More importantly, this concept is now advancing into clinical settings; for instance, the senolytic combination of dasatinib and quercetin (D+Q) is currently being evaluated in phase II clinical trials (e.g., NCT06355037) to reverse acquired resistance in advanced solid tumors like triple-negative breast cancer. As TIS appears to be a shared vulnerability across TKI-resistant cancers, senolytic strategies may represent a promising therapeutic avenue that extends beyond RCC to other solid tumors with acquired TKI resistance.

## Conclusions

In conclusion, our study found that resistant cells exhibited senescence-like phenotype without cell cycle arrest, allowing them to evade apoptosis and remain proliferative under pazopanib treatment. Transcriptome sequencing revealed that ALYREF was highly expressed in resistant cells and tumor models, closely associated with the bypass of cell cycle arrest. Mechanistically, ALYREF specifically recognizes the m^5^C modification on CCNA1, enhancing its mRNA stability and promoting Cyclin A1 expression. Elevated Cyclin A1 binds to CDK2, driving the G1-S transition, while the Cyclin A1-CDK2 complex phosphorylates p21, causing its relocation from the nucleus to the cytoplasm, thereby blocking its inhibitory effect on cell cycle progression and ultimately contributing to pazopanib resistance.

## Methods

### Cell lines and cell culture

The RCC cell line OS-RC-2 (RRID: CVCL_1626) and 786-O (RRID: CVCL_1051) was were purchased from National Collection of Authenticated Cell Cutures (NCACC, Shanghai, China). Cells were cultured in RPMI 1640 medium containing 10% fetal bovine serum (Gibco, USA), penicillin (25 units/ml), streptomycin (25 g/ml), 1% L-glutamine. To establish pazopanib-resistant RCC cell lines, the parental 786-O and OS-RC-2 cells were subjected to stepwise exposure to increasing concentrations of pazopanib. This gradual dose-escalation approach led to the generation of resistant sublines, designated as 78RP and OSRP.

### Animal experiment

All procedures in the *in vivo* experiment conformed to the institutional guidelines and were approved by the Animal Research Ethics Committee of Zhejiang University (SRRSH202208083).

To obtain pazopanib-resistant subcutaneous cell derived xenograft (CDX-RP) model, OS-RC-2 cells mixed with Matrigel (1:1) were injected into the flanks of 4-6-week-old female BALB/c nude mice (SLAC-Shanghai Laboratory Animal Center). When the volume of xenograft reached 200 mm^3^ (Volume = a × b^2^/2, a represents long axis and b represent short axis), mice were orally treated with vehicle or pazopanib (30mg/kg, twice a day, MedChemExpress) for 4 weeks. Following initial treatment, the fastest-growing tumors under pazopanib gavage were selected and dissected into 1 mm³ tissue fragments, which were then subcutaneously transplanted into the flanks of 4-6-week-old BALB/c nude mice to establish the next generation of xenografts. Mice were subsequently treated with either vehicle or pazopanib. CDX model treated with pazopanib from the 3rd generation xenografts were isolated and confirmed to be pazopanib-resistant (termed as CDX-RP), and the CDX models treated with vehicle for three generations were termed as CDX-WT.

For the renal orthotopic implantation model, 2 × 10^6^ OSRP cells were suspended in a 50 µL mixture of PBS and Matrigel with a 1:1 ratio and subsequently injected under the renal capsule of 5 weeks female NCG mice (GemPharmatech). After 4 weeks, mice were anesthetized and an *in vivo* imaging system (IVIS) was used to detect tumor growth twice a week. For pazopanib monotherapy, mice were administered pazopanib (30 mg/kg, twice daily) or vehicle via oral gavage. For ABT-263 monotherapy, mice received ABT-263 (100 mg/kg, once daily) or vehicle by oral gavage. In the combination treatment group, mice were treated with both pazopanib (30 mg/kg, twice daily) and ABT-263 (100 mg/kg, once daily) via oral gavage.

### Cell transfection

Short interfering RNA (siRNA) sequences were directly synthesized (GenePharma). The siRNAs were transfected into cells using Lipofectamine RNAiMAX transfection reagent (Invitrogen) according to manufacturer's guidance. Ectopic expression plasmids of indicated genes were synthesized by Yoche Biotechnology and transfected using jetPRIME (Polyplus-transfection). Lentivirus was synthesized by GENECHEM (Shanghai, China), and infected RCC cells with 5mg/mL polybrene for 3 days according to manufacturer's instruction. Stable infected cell lines were selected using puromycin (Selleck, Shanghai, China). The shRNA and siRNA sequences are listed in Table S1(Supporting Information).

### RNA isolation and quantitative real-time PCR (qRT-PCR)

TRIzol reagent (CWbiotech) was used to lyse cells and extract total RNA according to the manufacturer's instructions. qRT-PCR was performed using a 2× SYBR Green qPCR master mix (CWbiotech) and primers. The detailed primer sequences used in the study are listed in Table S2 (Supporting Information).

### Western blotting

Cells or tissue samples were lysed using RIPA lysis buffer (FDBio), and total protein was denatured at 98 °C for 20 minutes. Protein was then separated on 8-12% SDS-PAGE gels and transferred onto PVDF membranes. The membranes were incubated with primary antibodies at 4 °C for 12-16 hours, followed by incubation with appropriate HRP-conjugated secondary antibodies (Jackson ImmunoResearch). Detailed information on the primary antibodies used in this study is provided in Table S3 (Supporting Information).

### CCK8, colony formation, EdU assays

CCK8 assay, RCC cells suspended in RPM1640 (10% FBS) were seeded into 96-well dish at a density of 2000 cells per well and were treated with pazopanib or DMSO after cell attachment. The viability of RCC cells was determined by Cell Counting Kit 8 (YEASEN) and measured at OD 450 nm with the BioTek Gen5 system (BioTeck).

Colony formation assay, RCC cells suspended in RPM1640 (10% FBS) were seeded into 6-well dish at a density of 2500 cells per well and were treated with pazopanib or DMSO after cell attachment. After 2 weeks, colonies were counted using Image J software (NIH Image). The colonies with >50 cells under microscope were counted. Three different independent experiments were performed.

EdU assay, EdU staining proliferation kit was purchased from Meilunbio. The plates were added with EdU solution and were incubated for 2 h and then treated with 4% formaldehyde. After the process, the cells were stained with hochest and apollo solution and performed as the instruction described by Zeiss Axio Observer A1 Inverted Phase Contrast Fluorescence Microscope (ZEISS).

### Flow Cytometry Assay

For cell cycle assay, the RCC cells were collected and washed twice. Then, the cells were stained with PI staining solution (liankebio). After incubation for 20 minutes, the cell cycle of RCC cells was detected using flow cytometry.

For apoptosis assay, the transfected RCC cells were collected and washed for twice. Then, the cells were stained with annexin APC and PI staining solution (liankebio). After incubation for 5 minutes, the apoptosis rate of RCC cells were determined using flow cytometry (Beckman Coulter).

### SA-β-Gal assay

For RCC staining, cells were plated into 6-well tissue culture plates. SA-β-Gal staining was performed using the Senescence β-Galactosidase Staining Kit (Beyotime) according to the manufacturer's instructions. Images were quantified from 3 independent fields from 3 biological replicates using Image J software and the Image J cell counter tool.

For tumor tissue staining, tumor was flash frozen in Optimal Cutting Temperature (OCT) and 10 μm sections cut. Immediately after sectioning, samples were fixed and stained overnight with an X-Gal solution using a commercial kit (Servicebio), followed by incubation at 37 °C for 16-18 hours. After staining, sections were rinsed with PBS and distilled water, counterstained with Nuclear Fast Red (Servicebio) for 3 minutes, washed, dehydrated through graded ethanol, cleared in xylene, and mounted with neutral balsam. Images were quantified from 3 independent fields from 3 biological replicates using Image J software and the Image J cell counter tool.

### H&E and immunohistochemical (IHC) staining

Tissues were fixed in 10% (v/v) formaldehyde in PBS, embedded in paraffin, and cut into 5 μm sections and used for H&E staining and IHC staining with specific primary antibodies. To enhance antigen exposure, the slides were treated with 1 × EDTA at 98°C for 10 minutes for antigen retrieval. The slides were incubated with endogenous peroxidase blocking solution, and then were incubated with the primary antibody at 4 ℃ overnight. After rinsing with Tris-buffered saline, the slides were incubated for 45 minutes with biotin-conjugated secondary antibody, washed, and then incubated with enzyme conjugate horseradish peroxidase (HRP)-streptavidin. Freshly prepared DAB (Servicebio) was used as substrate to detect HRP. Finally, slides were counter-stained with hematoxylin and mounted with aqueous mounting media. Positive cells were calculated as the number of immunopositive cells × 100% divided by total number of cells/fields in 10 random fields at 400 × magnification. The slides were reviewed and scored by an experienced pathologist without the knowledge of patient outcome. Images were quantified from 3 independent fields from 3 biological replicates using Image J software and the Image J cell counter tool.

### Cytokine antibody array

Cytokine profiling was performed using the Quantibody® Human Cytokine Antibody Array 640 (RayBiotech), which enables quantitative detection of 640 human cytokines simultaneously. Briefly, samples were incubated on glass slide-based antibody arrays pre-spotted with cytokine-specific capture antibodies. After blocking and sample incubation, arrays were sequentially incubated with a biotinylated antibody cocktail and Cy3-conjugated streptavidin. Slides were scanned using an Agilent laser scanner, and fluorescence intensity was quantified. Cytokine concentrations were calculated based on standard curves using Q-Analyzer software (RayBiotech), following the manufacturer's instructions.

### RNA sequencing

Total RNA was extracted with TRIzol reagent (CWBio) following the manufacturer's instructions. Following the extraction of total RNA, mRNA was isolated from total RNA utilizing Dynabeads Oligo (dT) (Thermo Fisher, CA, USA). Following purification, the mRNA was fragmented into short fragments. Then, the RNA was used for sequencing library preparation (NEB, USA). Sequencing data was collected using the Illumina Novaseq 6000 (LC-Bio Technology CO., Ltd., Hangzhou, China) following the vendor's recommended protocol.

### ALYREF RIP ceRNA array

The RIP assays were performed by using Magna RIP Kit (Millipore, USA) according to the manufactures' guidelines. Briefly, 2 × 10^7 RCC cells were harvest and lysed in RIP lysis buffer. After centrifuged at 4 °C, the supernatant was incubated with anti-ALYREF antibodies and negative control IgG at room temperature. Then, the beads-antibody complex was washed and incubated with Proteinase K buffer. The immunoprecipitated RNA was purified using the QIAGEN RNeasy Kit. A total of 250 ng purified RNA was used for amplification and labeling. First-strand cDNA was synthesized using the AffinityScript RT Kit with a promoter primer, followed by second-strand synthesis using an anti-sense promoter. T7 RNA polymerase was then added to generate amplified cRNA from the double-stranded cDNA. Cyanine-3-CTP (Cy3) was incorporated for labeling, and labeled cRNA was purified again using the QIAGEN RNeasy Kit. Hybridization was performed at 65 °C for 17 hours with continuous rotation, and slides were subsequently washed and scanned using the Agilent Scanner G5761A (Agilent Technologies). Raw images were processed with Feature Extraction software (version 12.0.3.1, Agilent Technologies) to extract signal intensities. Data were then normalized using quantile normalization and further processed in GeneSpring software (version 14.8, Agilent Technologies). Probes were filtered by detection flags, retaining those marked as “Detected” in at least 80% of samples in any comparison group. Differentially expressed genes were identified using Student's t-test, with a cutoff of p ≤ 0.05 and fold change ≥ 2.

### mRNA stability assay

To analyze the stability of CCNA1 mRNA, stable cells were incubated with 5 μg/mL actinomycin D and collected at indicated time points after treatment. Total RNA was extracted and the half-time of the remaining CCNA1 mRNA was analyzed by qRT-PCR.

### Luciferase reporter assay

The wildtype and mutant form of CCNA1 was amplified and subcloned into the pGL3-basic backbone (Detailed sequence see Table S4). For the luciferase assay, cells were plated in 24-well plates and co-transfected with dual-luciferase reporter (WT or MUT) and ALYREF overexpression plasmid or short interfering RNA using Lipofectamine 2000 (Invitrogen) according to the manufacturer's instruction. Luciferase activity was measured by Dual-Luciferase Assay (YEASEN, Shanghai, China) according to the manufacturer's manual and Renilla luciferase activity was normalized against Firefly luciferase activity.

### Nuclear and cytoplasmic protein extraction

Nuclear and cytoplasmic proteins were extracted using a hypotonic buffer-based protocol. Briefly, cells were harvested and washed with pre-chilled PBS. For total protein extraction, cells were directly lysed in RIPA buffer with loading buffer. For subcellular fractionation, cell pellets were resuspended in ice-cold hypotonic buffer (20 mM Tris-HCl pH 7.4, 10 mM NaCl, 3 mM MgCl₂, supplemented with protease inhibitors) and incubated on ice for 15 minutes. Equal volume of 1% NP-40 was added, followed by vortexing for 15 seconds. The lysate was centrifuged at 4,000 rpm for 10 minutes at 4 °C to separate the cytoplasmic fraction (supernatant), which was collected and mixed with loading buffer. The nuclear pellet was washed four times with hypotonic buffer, then lysed in RIPA buffer supplemented with loading buffer. All samples were denatured by boiling and subjected to downstream analysis.

### Immunofluorescence (IF) assay

About 2 × 10^5^ RCC cells were seeded into 24-well plates. After adherence, the RCC cells were washed and fixed for 15 min. After washed for 3 times, the RCC cells were treated with 0.2% Triton X-100 for 10 min. Then, the cells were washed with PBS and blocked using 5% BSA for an hour. Then, the cells were incubated with primary antibodies p21 (1:200) at 4 °C overnight. The next day, the RCC cells were washed for 3 times and incubated with the fluorescent secondary antibodies (Invitrogen) for an hour at room temperature. Finally, the RCC cells were stained with DAPI for 10 min. The stained cells were photographed using confocal microscopy (Olympus fv3000).

### Statistical analysis

Results in the study were presented as the mean ± SD and were analyzed using GraphPad prism8 (GraphPad Software). The statistical difference between the two groups was measured by a two-tailed Student's t-test. Statistical significance was defined as * P value < 0.05, ** P value < 0.01, *** P value < 0.001, ****P value < 0.0001.

## Supplementary Material

Supplementary figures and tables.

## Figures and Tables

**Figure 8:**
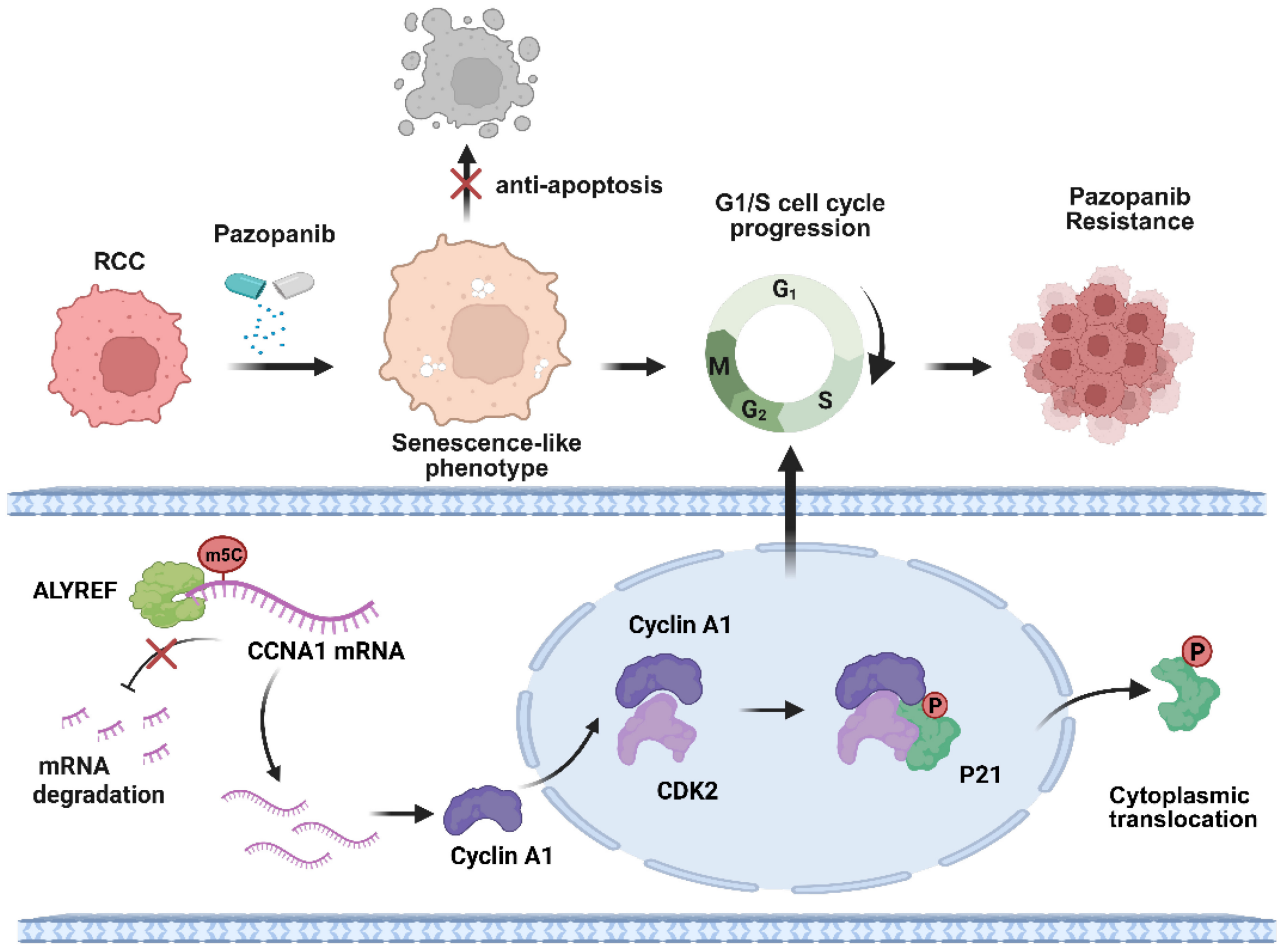
** Diagram of the Proposed Molecular Mechanisms for Pazopanib Resistance.** Schematic of the proposed molecular mechanisms for ALYREF-CCNA1 axis involvement in RCC pazopanib resistance. Image created with BioRender.com, with permission.

## Data Availability

The datasets supporting the conclusions of this article are available in the National Genomics Data Center, China National Center for Bioinformation / Beijing Institute of Genomics, Chinese Academy of Sciences (HRA014049, https://ngdc.cncb.ac.cn/gsa-human/) that are available upon reasonable request[49, 50].

## Abbreviations

RCC: Renal cell carcinoma
mRCC: Metastatic renal cell carcinoma
TKI: Tyrosine kinase inhibitor
RTK: Receptor tyrosine kinase
VEGFR: Vascular endothelial growth factor receptor
PDGF: Platelet-derived growth factor
ALYREF: Aly/REF export factor
m^5^C: 5-methylcytosine
m⁶A: N6-methyladenosine
CCNA1: Cyclin A1
CDK2: Cyclin-dependent kinase 2
p21 (CDKN1A): Cyclin-dependent kinase inhibitor 1A
p16 (CDKN2A): Cyclin-dependent kinase inhibitor 2A
SASP: Senescence-associated secretory phenotype
TIS: Therapy-induced senescence
SA-β-Gal: Senescence-associated β-galactosidase
AML: Acute myeloid leukemia
CDX: Cell-derived xenograft
EdU: 5-Ethynyl-2′-deoxyuridine
IHC: Immunohistochemistry
RIP: RNA immunoprecipitation
qRT-PCR: Quantitative real-time polymerase chain reaction
HHT: Homoharringtonine
PFS: Progression-free survival
ORR: Objective response rate
RB: Retinoblastoma protein
