# Supplementary material for: ALYREF-mediated m5C modification of CCNA1 drives escape from cell-cycle arrest and contributes to pazopanib resistance in Renal Cell Carcinoma
Source: Int J Biol Sci. 2026 Jun 17;22(12):6319–37. doi: 10.7150/ijbs.131368 (PMC13412283; doi:10.7150/ijbs.131368)
Supplement: Supplementary file 1 — Supplementary figures and tables. [file ijbsv22p6319s1.pdf]

Supporting Information

ALYREF-mediated m^5C modification of CCNA1 drives escape from cell-cycle arrest and contributes to pazopanib resistance in Renal Cell Carcinoma

Zeyi Lu, Yang Li, Ziwei Zhu, Fan Li, Yiming Ding, Lifeng Ding, Ruyue Wang, Yudong Lin, Wenqin Luo, Xudong Mao, Haohua Lu, Yejinpeng Wang, Meng xuan Li, Yuanlei Chen, Zhehao Xu, Yi Lu, Qiming Zheng, Haiyun Xie, Zhenwei Zhou, Liqun Xia*, Gonghui Li*, Mingchao Wang*

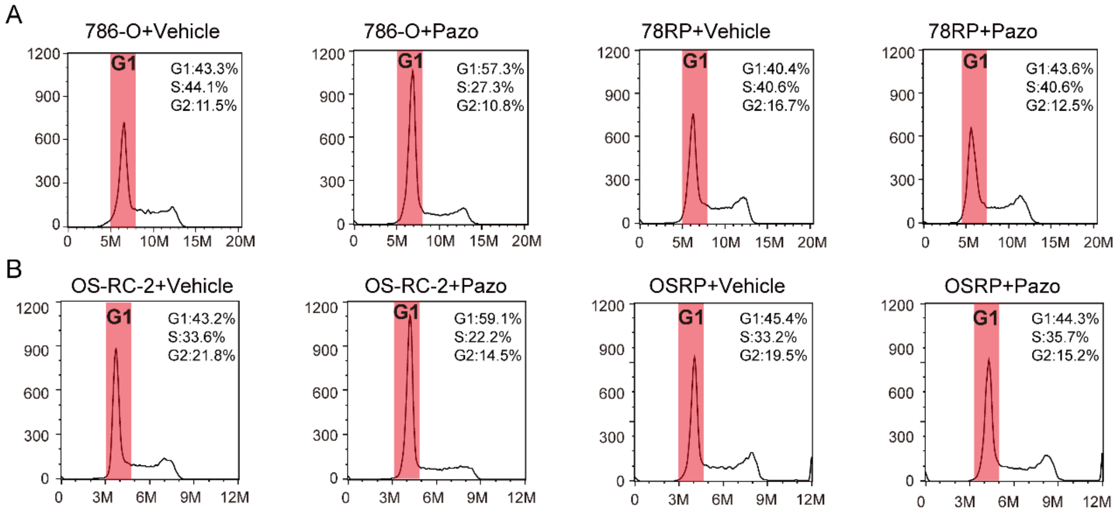

Figure.S1 Cell cycle profiling of Pazopanib-resistant cell lines and control cell lines with pazopanib treatment

(A) Flow cytometric analysis of cell cycle in Pazopanib-resistant cell lines and control cell lines with pazopanib treatment.

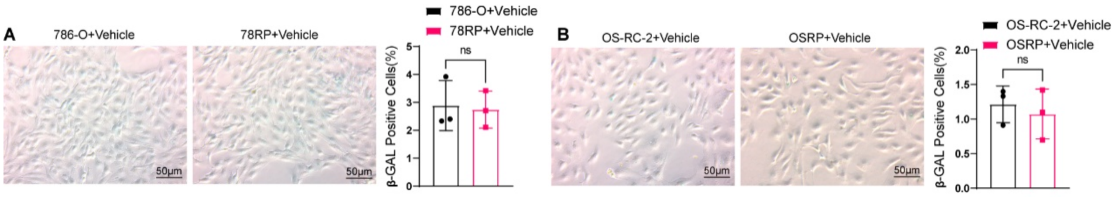

Figure.S2. SA-β-Gal stain in wild-type and pazopanib-resistant cell lines under vehicle-treated conditions.

(A, B) Representative images of SA-β-Gal staining and quantification in 786-O vs. 78RP and OS-RC-2 vs. OSRP cells under vehicle-treated conditions. Scale bar, 50μm. Data are presented as mean ± SD; ns, not significant.

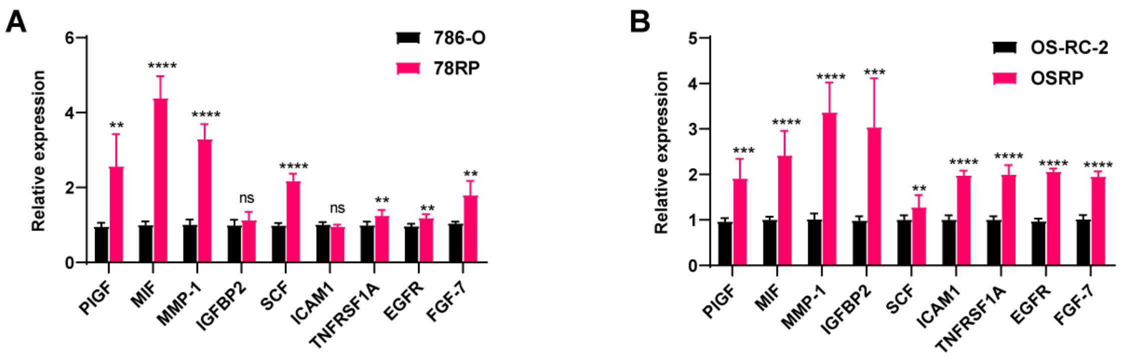

Figure.S3. Validation of SASP-associated cytokines in pazopanib-resistant cell lines.

(A, B) Quantitative analysis of representative upregulated cytokines (PlGF, MIF, MMP-1, IGFBP2, SCF, ICAM1, TNFRSF1A, EGFR, and FGF-7) in 786-O vs. 78RP and OS-RC-2 vs. OSRP cells. Data are presented as mean ± SD, **P < 0.01, ***P < 0.001, ****P < 0.0001; ns, not significant.

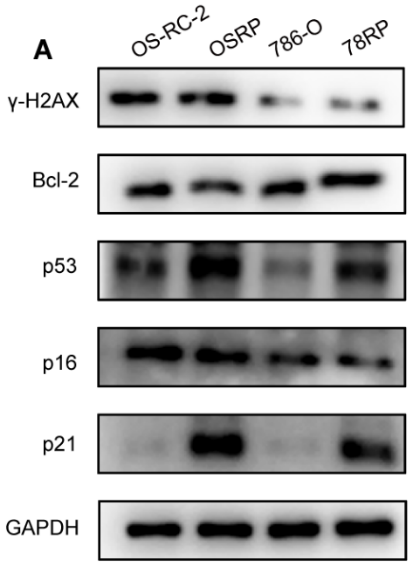

Figure.S4. Western blot analysis of senescence-associated markers in wild-type and pazopanib-resistant cell lines under vehicle treatment.

(A) Western blot analysis of γ-H2AX, Bcl-2, p53, p16, and p21 expression in wild-type (786-O, OS-RC-2) and pazopanib-resistant (78RP, OSRP) cell lines under vehicle-treated conditions.

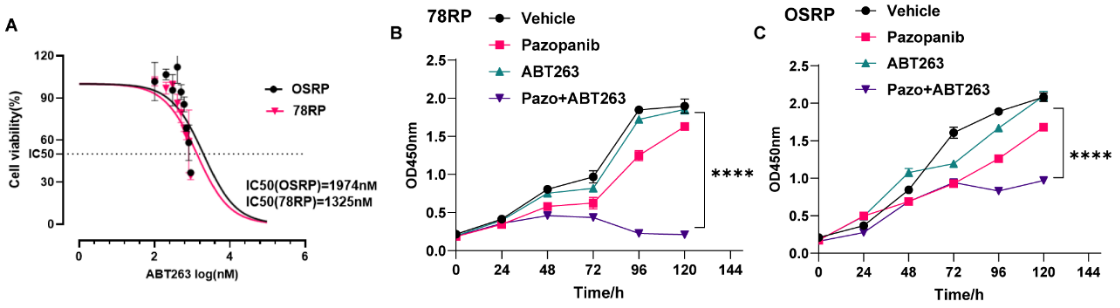

Figure.S5 Cytotoxic effect of ABT-263 and its combination with pazopanib in resistant cell lines.

(A) Dose-response curves of ABT-263 as a single agent in 78RP and OSRP cells, with IC50 values indicated. **(B, C)** Time-course CCK-8 assays of 78RP and OSRP cells treated with vehicle, single-agent pazopanib, single-agent ABT-263, or the combination of pazopanib and ABT-263. Data are presented as mean ± SD, ****P < 0.0001.

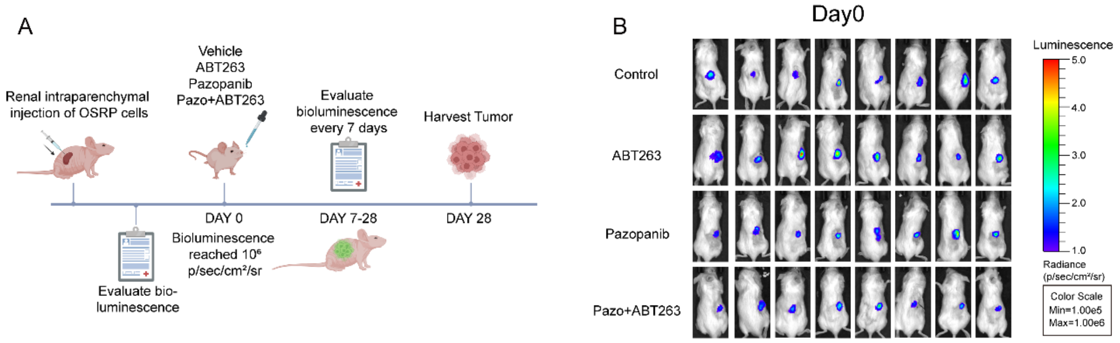

Figure.S6 Experimental design and baseline bioluminescence of orthotopic RCC model.

(A) Experimental schematic of orthotopic xenograft model establishment and treatment strategy. **(B)** Representative bioluminescence imaging of orthotopic renal tumor-bearing mice on day 0 before treating with vehicle, ABT263(100mg/kg, once a day), pazopanib (30mg/kg, twice a day), or the combination of ABT263 and pazopanib.

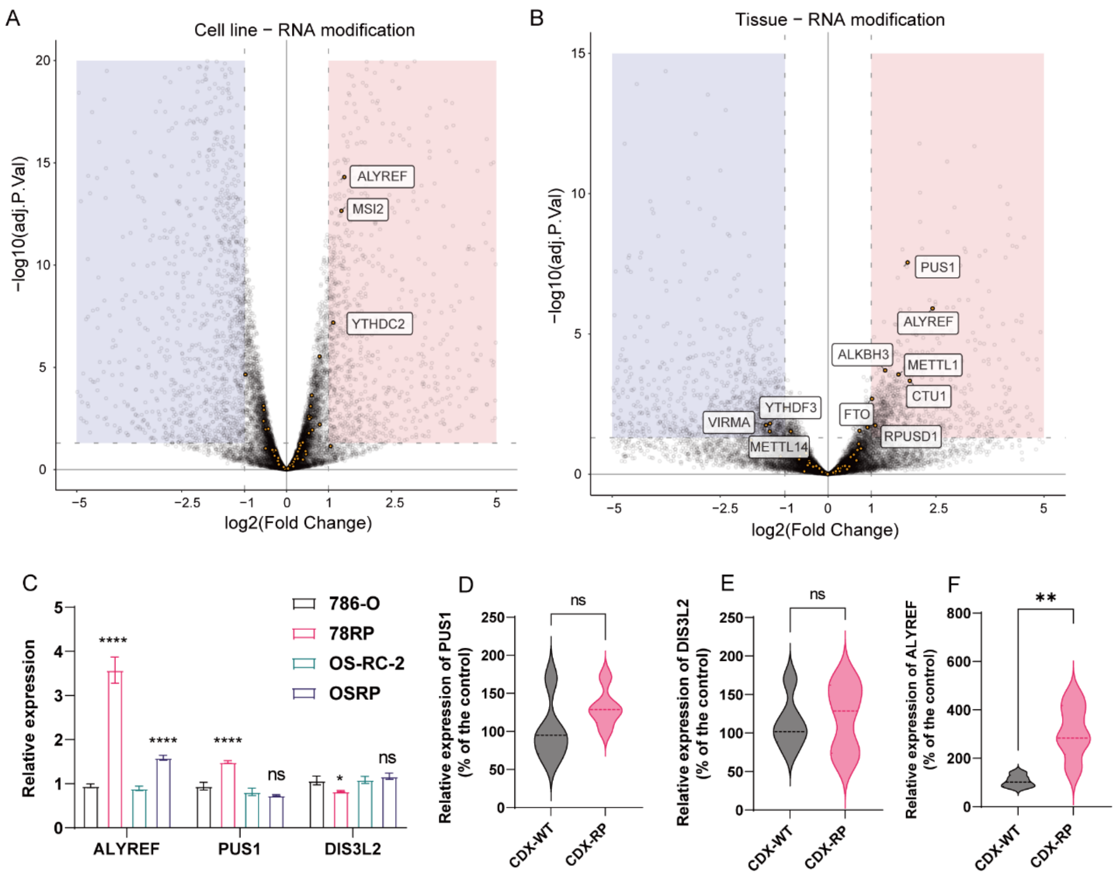

Figure.S7 RNA modification-related genes in resistant cells and tissues

(A, B) Volcano plots respectively showing differentially expressed genes which are related to RNA modification in resistant cells (A) and tissues (B). Notable RNA modification-related genes were identified with a threshold of absolute $\log_2$(fold change) ≥ 1 and adjusted P < 0.05. **(C)** RT-qPCR analysis of ALYREF, PUS1, and DIS3L2 expression in wild-type (786-O, OS-RC-2) and pazopanib-resistant (78RP, OSRP) cell lines. **(D, E)** RT-qPCR analysis of PUS1 and DIS3L2 expression in CDX-WT and CDX-RP tumor tissues. **(F)** RT-qPCR analysis of ALYREF expression in CDX-WT and CDX-RP tumor tissues. Data are presented as mean ± SD, *P < 0.05, **P < 0.01, ****P < 0.0001; ns, not significant.

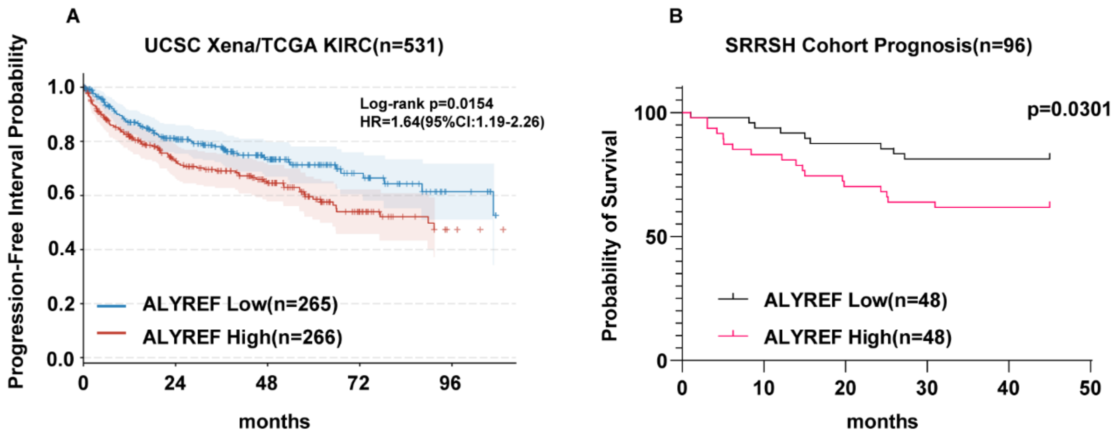

Figure.S8. Prognostic significance of ALYREF expression in RCC patients. **(A)** Kaplan–Meier survival curves of low and high ALYREF expression groups in TCGA KIRC cohort(n=531). **(B)** Kaplan–Meier survival curves of low and high ALYREF expression groups in SRRSH cohort(n=96).

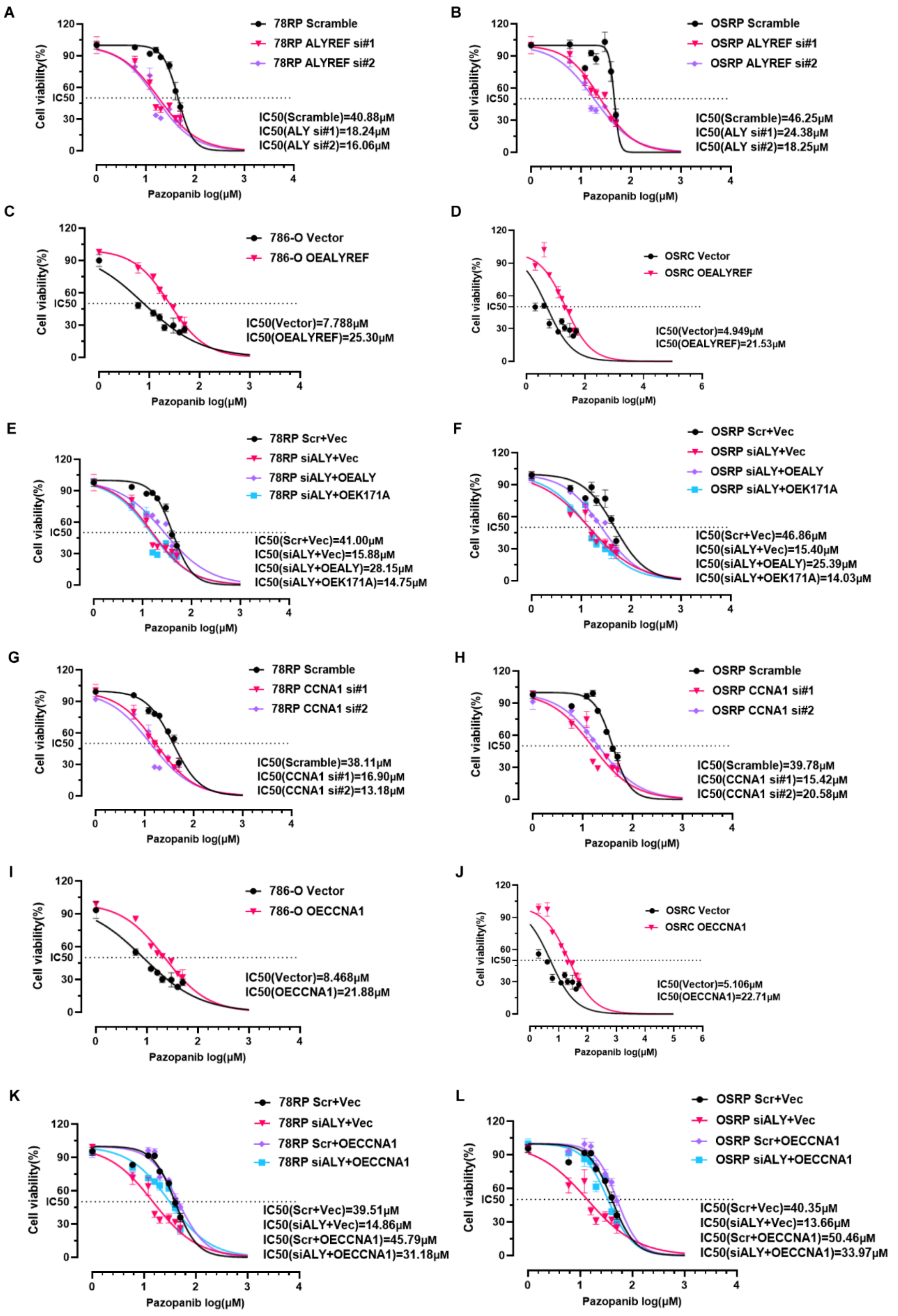

Figure.S9. IC50 values of pazopanib across experimental groups.

(A, B) Dose-response curves of pazopanib in 78RP and OSRP cells following ALYREF knockdown. **(C, D)** Dose-response curves of pazopanib in 786-O and OS-RC-2 cells following ALYREF overexpression. **(E, F)** Dose-response curves of

pazopanib in 78RP and OSRP cells following ALYREF knockdown and rescue with either wild-type ALYREF or m^5C-binding-deficient mutant (K171A). **(G, H)** Dose-response curves of pazopanib in 78RP and OSRP cells following CCNA1 knockdown. **(I, J)** Dose-response curves of pazopanib in 786-O and OS-RC-2 cells following CCNA1 overexpression. **(K, L)** Dose-response curves of pazopanib in 78RP and OSRP cells following ALYREF knockdown and rescue with CCNA1 overexpression. IC50 values are indicated for each group.

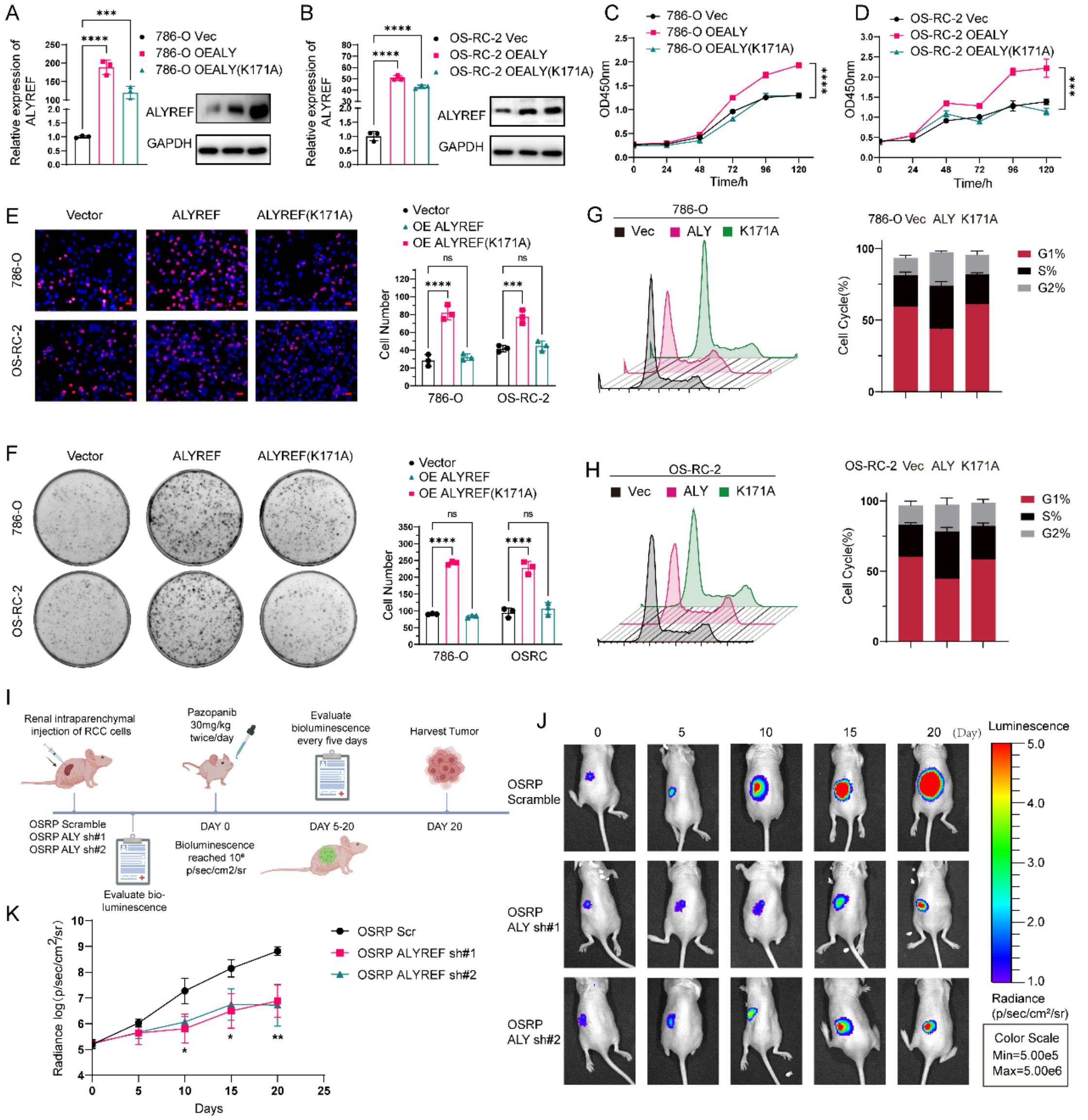

Figure.S10. ALYREF Drives Pazopanib Resistance Through Cell-Cycle Regulation.

(A, B) qPCR and Western blot analysis of ALYREF expression in wild-type renal cancer cells (786-O and OS-RC-2) transfected with either wild-type ALYREF (OEALY) or its m^5C recognition site mutant (OEALY-K171A). **(C, D)** CCK8 assay of 786-O and OS-RC-2 following overexpression of ALYREF or its m^5C-binding-deficient mutant (K171A), measured at the indicated time points. **(E)** Representative images of EdU assay and its quantification data of 786-O and OS-RC-2 following overexpression of ALYREF or its m^5C-binding-deficient mutant (K171A). Scale bar, 20μm. **(F)** Representative images of colony-formation assay and its quantification data of 786-O and OS-RC-2 following overexpression of ALYREF or its m^5C-binding-deficient mutant (K171A). **(G, H)** Flow cytometric analysis of cell cycle and its

quantification data of 786-O and OS-RC-2 following overexpression of ALYREF or its m^5C-binding-deficient mutant (K171A). **(I)** Experimental schematic of orthotopic xenograft model establishment and treatment strategy. **(J)** Representative bioluminescence images of mice bearing orthotopic OSRP tumors at indicated time points. **(K)** Quantification of in vivo tumor burden based on bioluminescent signals signal intensity. Data are presented as mean ± SD, *P < 0.05, **P < 0.01, ***P < 0.001, ****P < 0.0001; ns, not significant.

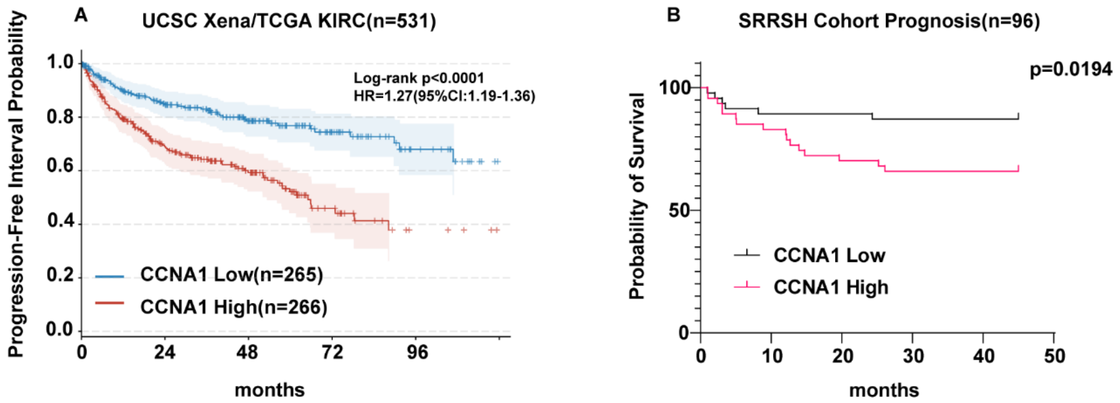

Figure.S11. Prognostic significance of CCNA1 expression in RCC patients. **(A)** Kaplan–Meier survival curves of low and high CCNA1 expression groups in TCGA KIRC cohort(n=531). **(B)** Kaplan–Meier survival curves of low and high CCNA1 expression groups in SRRSH cohort(n=96).

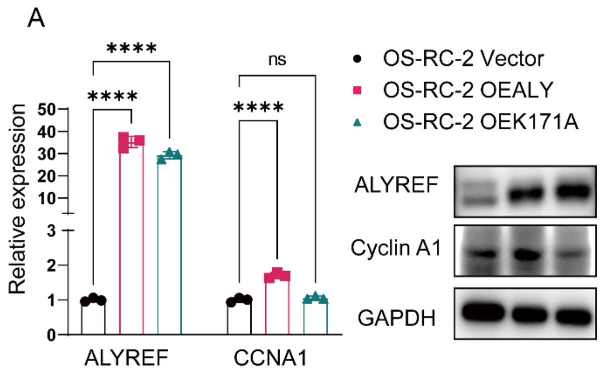

Figure.S12. CCNA1 Functions as a Key Downstream Effector of ALYREF.

(A) qPCR and Western blot analysis of ALYREF and CCNA1 expression in OS-RC-2 cells overexpressing wild-type or mutant ALYREF.

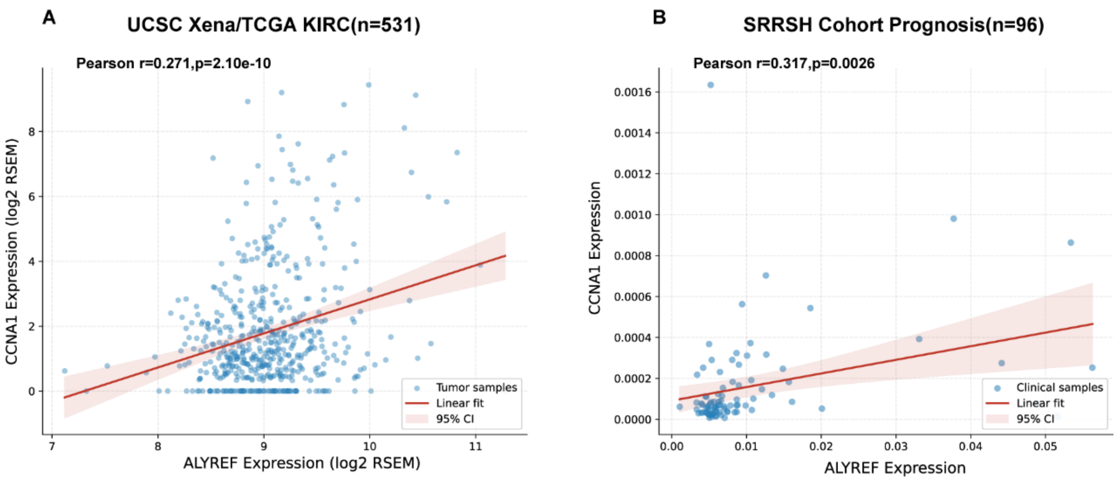

Figure.S13. Correlation between ALYREF and CCNA1 expression in RCC patients.

(A) Correlation analysis of ALYREF and CCNA1 expression in TCGA KIRC cohort (n=531). **(B)** Correlation analysis of ALYREF and CCNA1 expression in SRRSH cohort (n=96).

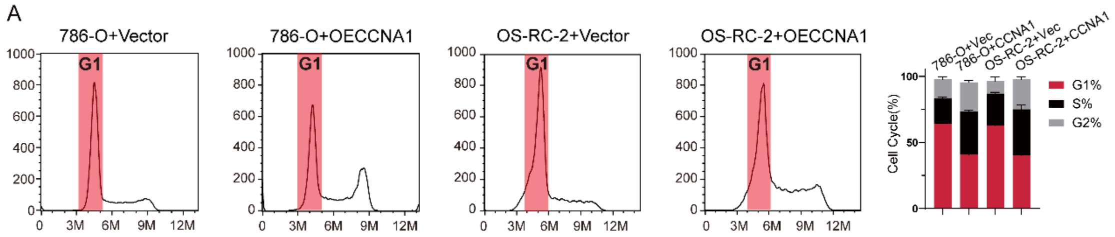

Figure.S14. CCNA1 plays a role in promoting cell cycle progression.

(A) Flow cytometric analysis of cell cycle and its quantification data of 786-O and OS-RC-2 following CCNA1 overexpression.

Table S1

The Transcripts Per Million (TPM) value of RNA modification related genes in RNA-seq data of 78RP (R1-3) and 786-O (S1-3).

	S1	S2	S3	R1	R2	R3
YTHDF3	81.51434	63.11861	64.08194	84.94087	95.77145	77.65666
YTHDF2	122.6659	99.46407	94.78911	108.9214	113.7935	92.07798
YTHDF1	97.83978	71.33471	65.99294	105.6608	119.5971	110.7808
YTHDC2	6.857593	5.133799	5.430481	11.10288	16.76655	14.96263
YTHDC1	0.702468	0.768777	1.564536	2.866848	3.508884	0.912333
YBX1	1705.27	1770.075	1918.016	2048.237	1947.029	2021.523
WTAP	55.18997	49.25946	56.12924	42.00007	48.34777	36.09419
WBSCR22	315.6753	336.0306	357.0264	360.7558	359.8068	391.6621
TUT1	40.0954	44.0627	47.14905	45.99939	34.64795	42.4562
TET2	2.308856	1.926133	2.003487	1.384216	1.793398	1.838125
RPUSD4	29.89755	35.13297	34.00935	48.50806	52.98366	47.1614
RPUSD3	28.94787	37.29191	33.00975	31.63503	31.26124	35.78283
RPUSD2	30.16643	33.14621	24.14096	16.19902	17.34847	17.11493
RPUSD1	79.83169	84.37524	70.29524	86.88846	121.3533	111.397
RNMT	20.55871	18.8575	22.18792	21.9693	26.46618	23.7503
RBM15	23.74678	24.00784	23.86052	18.96582	24.81908	22.03533
PUS7L	4.388463	2.549371	3.099976	6.17864	6.000287	5.200538
PUS7	34.26515	25.76856	26.98161	42.27066	56.90594	45.21113
PUS3	10.19899	9.121161	7.999789	11.50948	16.63702	14.80996
PUS10	2.066032	1.720368	1.511699	2.721298	2.673355	2.515563
PUS1	36.84132	37.47931	28.69209	55.35014	61.7383	52.96276
PRRC2A	13.79229	11.91343	17.98122	17.22228	16.27773	14.86515
PCIF1	67.44543	81.49327	82.65003	95.15587	86.80969	110.5481
NSUN6	10.48432	4.208146	5.783698	8.398155	7.031421	6.521121
NSUN5	62.88156	77.25704	70.9007	59.75007	53.50126	58.33677
NSUN4	15.62536	14.89977	12.74755	28.05938	30.4166	26.72046
NSUN3	3.680557	2.892294	2.388501	4.887529	4.361598	4.693225
NSUN2	213.3442	169.5178	153.7039	264.1011	234.9776	185.6843
MSI2	4.46283	2.894751	3.486916	9.462418	10.69664	10.23116
METTL5	35.34553	39.21524	37.90844	38.75862	44.87504	47.92049
METTL3	46.86065	46.27918	43.36635	35.52405	44.2044	35.99085
METTL16	18.21859	14.83832	15.28267	21.2704	24.02093	21.16965
METTL14	9.246686	5.602864	6.270674	6.66966	8.356375	6.079642
METTL1	51.75704	59.15367	46.5092	67.38733	61.36436	52.06228
IGF2BP3	24.80005	19.6353	23.6982	33.02929	42.64189	39.92883
IGF2BP2	85.40305	83.39037	84.20594	121.3437	166.8396	151.3245
IGF2BP1	0.10258	0.024405	0.087872	0.034315	0	0.213162
HNRNPA2B1	221.5506	186.0535	191.9643	236.1853	326.0284	289.1945
FTO	13.35506	10.57643	11.88878	9.75429	9.020558	9.122592
ELP3	21.97082	24.22729	21.33216	29.13922	21.90235	23.19009
DKC1	88.90804	82.97371	79.43394	99.92204	112.0335	93.7986
DIS3L2	13.31123	12.04563	12.81471	15.91652	19.18717	18.8329
CTU2	45.16185	46.51521	43.41199	39.59832	31.98707	33.95382

CTU1	22.07321	28.08161	28.39555	33.42173	25.04465	25.95179
ALYREF	136.0538	164.8138	115.1202	389.9866	419.2126	429.1065
ALKBH8	12.23689	5.378731	6.768844	15.85983	17.6001	13.96848
ALKBH5	201.3762	193.9238	203.6808	197.5944	175.3601	196.2605
ALKBH3	55.41656	48.88548	40.65528	39.85278	44.06156	46.57993
ALKBH1	19.95334	17.41994	19.71811	21.01091	22.12323	20.10062
ADAT3	7.579044	9.864266	8.911033	12.52747	11.13469	10.19072
ADAT2	8.442262	5.062113	6.467437	8.265606	6.9471	7.594867

Table S2

The Transcripts Per Million (TPM) value of RNA modification related genes in RNA-seq data of CDX-RP (A1-3) and CDX-WT (B1-3).

	B1	B2	B3	A1	A2	A3
YTHDF3	36.32189	116.9141	68.76719	17.8606	21.99399	30.86074
YTHDF2	93.24072	128.5055	107.1249	72.58846	87.13749	102.9032
YTHDF1	45.33378	96.94296	64.10511	43.00917	61.81185	62.56261
YTHDC2	14.44597	21.65408	13.48084	12.87021	12.68638	13.96531
YTHDC1	11.63012	1.379458	2.213271	3.676605	8.144797	2.58344
YBX1	486.4653	794.7713	1234.589	475.7245	518.5889	540.0172
WTAP	58.37089	69.21688	85.12826	27.85387	27.86458	42.0295
WBSCR22	169.2666	227.7683	351.8859	362.3706	352.579	252.8261
TUT1	24.14502	31.11122	38.08176	33.17834	29.42944	23.65598
TET2	2.694943	7.089485	3.657858	1.783381	2.52636	2.475567
RPUSD4	30.91877	33.56258	33.0521	19.38951	26.29103	32.97776
RPUSD3	12.94474	19.96001	18.19765	21.18433	19.28207	13.48309
RPUSD2	15.77173	17.77163	19.07968	24.86647	27.30631	20.85375
RPUSD1	39.66158	51.4269	68.74551	101.1498	96.11202	82.61203
RNMT	25.37516	41.02496	26.75712	10.38241	12.41762	15.40365
RBM15	12.70902	18.61137	12.13446	7.968722	10.79257	16.91001
PUS7L	5.535681	7.531024	5.182725	7.658971	8.337334	8.624553
PUS7	30.67753	28.35924	29.52739	13.34324	16.62369	28.85376
PUS3	16.97274	17.75984	13.30233	11.10864	12.15771	20.10534
PUS10	5.436218	4.259988	2.347163	2.457565	4.042357	5.621094
PUS1	22.17623	21.66163	25.96878	72.09883	71.07651	62.86967
PRRC2A	9.331249	17.62345	14.49173	1.117374	7.833445	2.440482
PCIF1	107.0845	72.23141	65.08219	88.02683	96.54313	107.3347
NSUN6	16.32894	26.21867	14.63525	11.65109	15.32605	14.98845
NSUN5	15.51308	35.99536	48.37569	41.33204	43.11692	29.99844
NSUN4	18.32206	28.64665	14.83304	10.02251	11.42179	16.42324
NSUN3	3.95686	8.147503	5.18097	3.449379	3.79328	2.720563
NSUN2	61.19615	82.6626	92.99067	36.35521	44.72923	65.51756
MSI2	8.339204	18.26009	8.735483	13.38149	16.13261	15.74787
METTL5	40.77936	36.70219	44.01608	35.686	42.91401	39.85724
METTL3	44.3331	45.61364	29.75491	23.5931	25.40743	32.38764
METTL16	15.53587	10.11946	16.21473	10.43549	9.103356	12.36663
METTL14	10.2515	23.15146	15.05704	3.093475	4.355454	8.241297
METTL1	53.5259	43.84338	47.55224	145.866	127.276	92.54467
IGF2BP3	12.04604	22.59436	16.3649	1.193306	2.05789	9.469653
IGF2BP2	54.36126	33.12314	34.06694	15.72782	19.8436	29.84085
IGF2BP1	0.194095	0.282016	0.372924	0.162694	0.198229	0.115486
HNRNPA2B1	217.0852	265.6079	289.9766	137.4906	141.1913	167.077
FTO	16.43825	16.69736	10.62121	23.78314	22.27137	26.25546
ELP3	17.88969	12.05315	11.77049	12.78301	14.85619	16.5773
DKC1	32.77113	25.52168	40.82678	41.82154	37.56843	45.99105
DIS3L2	9.439792	10.06228	10.701	17.69106	14.61704	14.12754
CTU2	21.36297	21.26254	22.77486	30.03007	31.11433	26.73779

CTU1	34.95095	20.98852	30.03348	112.2111	81.71768	67.83712
ALYREF	63.47206	44.61057	94.28435	336.5425	239.2757	334.2136
ALKBH8	7.579471	9.051646	6.738349	6.962463	7.210706	9.143133
ALKBH5	276.2979	219.598	229.069	149.032	173.3654	204.2677
ALKBH3	32.79175	29.99771	31.85852	63.53197	70.59007	60.59736
ALKBH1	19.04126	18.26268	15.83944	10.04425	8.449305	13.41315
ADAT3	7.802918	12.60695	11.88565	14.14172	15.50746	5.35379
ADAT2	2.922039	18.654	8.761017	2.939167	3.289335	5.048422

Table S3

siRNAs used in this study.

Gene Symbol	siRNA sequence (sense 5’→3’)
si-ALYREF#1	*CAGGAACUCUUUGCUGAAUTT*
si-ALYREF#2	*GAGGUGGCAUGACUAGAAATT*
si-CCNA1#1	*CUCCCAGUCUGAAGAUAUATT*
Si-CCNA1#2	*GCCUGAGUGAGCUUCAUAATT*

Table S4

Primers used for quantitative Real Time-PCR in this study.

Gene Symbol	Forward primer (5'→3')	Reverse primer (5'→3')
GAPDH	*GTCTCCTCTGACTTCAACAGCG*	*ACCACCCTGTTGCTGTAGCCAA*
ALYREF	*GCAGGCCAAAACAACTTCCC*	*AGTTCCTGAATATCGGCGTCT*
CCNA1	*TAGACACCGGCACACTCAAG*	*AGGAGAGATGAATCTACCAGCAT*
p21	*TGTCCGTCAGAACCCATGC*	*AAAGTCGAAGTTCCATCGCTC*
PUS1	*TGCTCATGGCCTATTCGGG*	*CGGACACACCCTTGTCTGT*
DIS3L2	*TCCCCGGATGGTGATCGAG*	*GGAAGCAGTTTCACGACCAC*
FGF1	*GCCCTGACCGAGAAGTTTAATC*	*GCCCTGACCGAGAAGTTTAATC*
LGALS3	*ATGGCAGACAATTTTTCGCTCC*	*GCCTGTCCAGGATAAGCCC*
ISG15	*CGCAGATCACCCAGAAGATCG*	*TTCGTCGCATTTGTCCACCA*
C1orf226	*CCTCCCATAACCAGAAAGCGA*	*TCTGTCCCATACTCCAGACTG*
FAM107A	*GCAGCGTGTCCTAGAGCAC*	*CCGCAGGTTTTCCCTGACT*
COX4I1	*GAGAAAGTCGAGTTGTATCGCA*	*GCTTCTGCCACATGATAACGA*
PAGE1	*ATCTATCGGCGTAGACCAATGA*	*TTCCACTTCGTCAGGTTGCTC*
HSBP1L1	*GCTCTGACGGCAACATTAAACC*	*AGCTTGCACCATTAAGTCCTTG*
KATNAL2	*AGTGCCAACTTCGGCCTAC*	*CTCAGAGGTTTCAGCAGTCGT*
NUDT6	*ATGCTTGCCCGAACCTACG*	*GAGATGCCCCCGAATCTGTC*
DDC	*TGGGGACCACAACATGCTG*	*TCAGGGCAGATGAATGCACTG*
SAA2	*GCTTCTTTTCGTTCCTTGGCG*	*GCCGATGTAATTGGCTTCTCTCA*

Table S5

Antibody used in this study.

Antibody	company	catalog number
ALYREF	*Cell Signaling Technology*	*12655S*
GAPDH	*Abcam*	*ab8245*
Cyclin A1	*Abcam*	*ab270940*
p21	*Cell Signaling Technology*	*2947S*
Bcl-2	*Cell Signaling Technology*	*15071S*
Histone H3	*Abcam*	*ab1791*
Phospho-p21(Thr57)	*Invitrogen*	*PA5-106197*
p53	*AR*	*ARH2042*
m5C	*Abcam*	*Ab10805*
p16	*Huabio*	*ET1608-62*
γ-H2AX	*Abclonal*	*AP0099*

Table S6

The detailed sequence of CCNA1 WT and CCNA1 MUT.

	Sequence
**NM_001413923-CCNA1 WT**	AGTTGTTCCGGACACATAGAAAGATAACGACGGGAAG<br>AGCGGGGCCCGCTTTGGGGTCCAGGCAGGTTTTGGGG<br>CCTCCTGTCTGGTGGGAGGAGGCCGCAGCGCAGCAC<br>CCTGCTCGTCACTTGGGATGGAGACCGGCTTTCCCGC<br>AATCATGTACCCATGTACTCACCAGAGCCCCGCTGGGC<br>CAGGATCCCCCGCAGAGGACAGTGCTAGGGCTGCTAA<br>CTGCAAATGGGCAGTACAGGAGGACCTGTGGCCAGG<br>GGATCACAAGAATCAGGTGTTATTCTGGATCAGAAAA<br>TGCCTTCCCTCCAGCTGGAAAGAAAGCACTCCCTGAC<br>TGTGGGGTCCAAGAGCCCCCCAAGCAAGGGT
**NM_001413923-CCNA1 MUT**	AGTTGTTCCGGACACATAGAAAGATAACGACGGGAAG<br>AGCGGGGCTCGCTTTGGGGTCTAGGCAGGTTTTGGGG<br>CCTCCTGTCTGGTGGGAGGAGGCCGCAGCGCAGCAC<br>CCTGCTCGTCACTTGGGATGGAGACCGGCTTTCCCGC<br>AATCATGTACCCATGTACTCACCAGAGCCCCGCTGGGC<br>CAGGATCCCCCGCAGAGGACAGTGCTAGGGCTGCTAA<br>CTGCAAATGGGCAGTACAGGAGGACCTGTGGCCAGG<br>GGATTACAAGAATCAGGTGTTATTCTGGATCAGAAAAT<br>GCCTTCCCTCCAGCTGGAAAGAAAGCACTCCCTGACT<br>GTGGGGTCCAAGAGCCCCCCAAGCAAGGGT
